# Identification and characterization of factors that govern apicoplast segregation in *Toxoplasma gondii*

**DOI:** 10.1038/s41467-026-78102-x

**Published:** 2026-10-05

**Authors:** Andrew J. Mead, Rebecca R. Pasquarelli, Brielle H. Wong, Isabelle Coppens, Jihui Sha, James A. Wohlschlegel, Peter J. Bradley

**Affiliations:** 1 Department of Microbiology, Immunology and Molecular Genetics, University of California, Los Angeles, Los Angeles, CA USA; 2 Department of Molecular Microbiology and Immunology, Johns Hopkins University Bloomberg School of Public Health, Baltimore, MD USA; 3 Department of Biological Chemistry and Institute of Genomics and Proteomics, University of California, Los Angeles, Los Angeles, CA USA; 4 Molecular Biology Institute, University of California, Los Angeles, Los Angeles, CA USA

**Keywords:** Chloroplasts, Parasite biology

## Abstract

The apicoplast is an essential plastid organelle of apicomplexan parasites that supports indispensable metabolic pathways, and its successful inheritance during cell division is critical for parasite survival. Here, we identify two previously uncharacterized proteins, APP1 and APP2, as key components of the apicoplast segregation machinery in *Toxoplasma gondii*. We show that APP1 is an inner membrane complex associated protein that is expressed during daughter bud development and localizes near the centrosomes and apicoplast. Loss of APP1 results in apicoplast loss and missegregation and growth defects without perturbing centrosome duplication or other organelles. Deletion mapping reveals regions of APP1 that are essential for apicoplast inheritance. Proximity labeling of APP1 further identified APP2, which localizes adjacent to APP1. APP2 is also found at the poles and along the length of the elongating apicoplast during organelle partitioning. Disruption of APP2 phenocopies loss of APP1, leading to lethal apicoplast inheritance defects. Interdependence analyses suggest that APP1 anchors the segregation machinery at the daughter IMC while APP2 associates with the apicoplast membrane, together forming a bridge that links the apicoplast to the centrosome during endodyogeny. These findings uncover a previously unknown molecular architecture controlling apicoplast segregation and highlight new parasite-specific targets for therapeutic intervention.

## Abstract (web summary)

Here, Mead et al. identify two apicoplast proteins, APP1 and APP2, in T. gondii, which are critical for anchoring the apicoplast to the centrosome and segregation machinery thereby facilitating its inheritance to the daughter cell during cell division.

## Introduction

T*oxoplasma gondii* (*T. gondii*) is an obligate intracellular parasite belonging to the phylum Apicomplexa, a diverse group that includes the human pathogens *Plasmodium spp*. and *Cryptosporidium spp*., as well as the veterinary pathogens *Neospora caninum* and *Eimeria spp*^1^. *T. gondii* infects nearly one-third of the global human population and causes significant disease in immunocompromised individuals and congenitally infected infants^2,3^. Pathogenesis of these organisms stems largely from rapid parasite replication by endodyogeny, a process in which two daughter buds form inside a maternal parasite^4^. The nascent daughters form on a scaffold of the inner membrane complex (IMC), a membrane-cytoskeletal organelle that connects the parasite’s subpellicular cytoskeleton with the plasma membrane. While micronemes, rhoptries, and daughter IMC are synthesized de novo in forming daughter buds, other organelles are replicated in the maternal parasite and segregated into the daughters.^5^ In the initial stages of endodyogeny, the centriole is duplicated, and then the Golgi apparatus, apicoplast, nucleus and endoplasmic reticulum, and finally the mitochondrion are segregated into the new daughters. Once the daughter cells have developed, the maternal IMC is degraded and partially recycled, and the daughter cells adopt the maternal plasma membrane to complete their replication cycle.

One parasite-specific organelle is the apicoplast, a four membrane bound, non-photosynthetic plastid that was acquired from the secondary endosymbiosis of a red algae which has been adapted for metabolic processes^6^. In *T. gondii*, the apicoplast supports several indispensable biosynthetic pathways including heme biosynthesis, fatty acid synthesis via the type II fatty acid synthesis (FASII) pathway, and the generation of isoprenoid precursors through the methylerythritol phosphate (MEP) pathway. In addition, the apicoplast contributes to the synthesis of lysophosphatidic acid, which is required for phospholipid production and membrane expansion during parasite replication. Collectively, the products of these pathways are essential for membrane biogenesis, mitochondrial electron transport, and protein trafficking, making the apicoplast indispensable for parasite survival^7^. Drugs that target the apicoplast typically result in a delayed death phenotype in which parasites lacking the organelle are able to complete an additional intracellular development cycle before perishing^8^. However, the bacterial origins of the apicoplast enable enhanced safety and selectivity profiles of anti-parasitic drugs, such as antibiotics like clindamycin or azithromycin, that target apicoplast translation with minimal host toxicity. Therefore, improving our understanding of apicoplast biogenesis processes promises to enable new avenues for the development of apicoplast-targeting compounds.

Another potential means of targeting the apicoplast lies in disrupting its segregation into daughter buds. Unlike plant plastids, which segregate independently from the nucleus, apicoplast segregation is coupled to nuclear division by linking the apicoplast to the machinery of nuclear division, the mitotic spindle and centrosomes^9^. During interphase, the circular apicoplast and centrosome reside at the apical side of the nucleus^5^. At the beginning of replication, the centrosome migrates to the basal side of the nucleus and duplicates, while the apicoplast deforms from a circular to an elongated morphology^10^. The duplicated centrosomes then return to the apical side of the nucleus, and the elongating apicoplast is re-associated with the centrosomes. The apicoplast next adopts a U-shaped morphology as the separate poles are brought into the two forming daughter buds. Finally, the apicoplast is constricted by dynamin related protein A (DrpA), as the centrosome and fissioned apicoplast are pulled into each new daughter cell, with each receiving a single apicoplast and centrosome^11^.

The segregation of the apicoplast is an actomyosin dependent process as depletion of MyoF prevents apicoplast and centrosome association, resulting in apicoplast inheritance defects^12^. MyoF is responsible for the dynamic branching and retracting process in which the tips or sides of the apicoplast associate with the centrosomes, allowing for the coupling of nuclear and apicoplast division^13^. In addition, the apicoplast is coated with ATG8, an autophagy-related protein that has been repurposed for associating the apicoplast and centrosome^14^. ATG8 binding partners associate with the protein primarily via ATG8-interacting motifs/LC3-interacting regions (AIMs/LIRs)^15^. AIMs in eukaryotes consist of WxxL motifs, which are conserved W/F/YxxL/I/V protein sequences, and xLIR motifs, which contain WxxL motifs but feature flanking amino acid residues that further support the interaction of a protein with ATG8. LIRs on ATG8-interacting proteins bind at the central LIR-docking site (LDS) of ATG8, where the LDS of ATG8 has been shown to be required for ATG8 localization to the apicoplast^16^. The lipidation of ATG8 with phosphatidylethanolamine (PE) is also shown to be critical for the localization of ATG8 to the apicoplast and subsequent apicoplast inheritance^17^. Localization of ATG8 to the apicoplast requires the upstream cytosolic maturation factors ATG3, 4, and 7^18,19^, the ATG5, 12, and 16L1 complex, and the vesicle fusion protein SNAP29^16^. ATG5-12-16L1 and SNAP29 localize at the interface of the elongating apicoplast and developing daughter IMC on peri-apicoplast vesicles carrying ATG8-PE. The ATG5-12-16L1 complex functions as an E3-like enzyme that lipidates ATG8, and SNAP29 mediates the fusion of vesicles and subsequent integration of ATG8-PE to the apicoplast outer membrane. Depletion of any of these autophagy proteins or SNAP29 results in mislocalization of ATG8 to the cytosol and defects in apicoplast inheritance.

Previously, we identified several IMC-associated proteins with unique localizations to both inner and outer faces of developing daughter buds (IAPs)^20^. We rationalized that IAPs localized to the inner face of the forming IMC may participate in apicoplast segregation due to their close proximity during replication. Therefore, we utilized TurboID proximity labeling of IAP3 and identified a novel protein, Apicoplast Partitioning Protein 1 (APP1), and directly demonstrated a role in apicoplast inheritance by gene knockout. We then used deletion analyses to identify regions of the protein that are important for localization and function. We extended our investigation by proximity labeling APP1 and identified a second novel protein, Apicoplast Partitioning Protein 2 (APP2), whose disruption also results in apicoplast-specific inheritance defects. Both APP1 and APP2 localize closely to the centrosome and apicoplast at the daughter IMC during apicoplast segregation. APP2 additionally forms punctate points along the length of the elongated apicoplast. When APP1 is disrupted, APP2 is enriched on the apicoplast and loses its daughter IMC localization. In contrast, when APP2 is disrupted, APP1 becomes associated with the developing daughter IMC and loses its apicoplast localization. We therefore report that APP1 and APP2 function as novel factors involved in the association of the apicoplast to the centrosome.

## Results

### IAP3 is an IMC and apicoplast localizing daughter bud protein whose loss results in growth defects

**Fig. 1:**
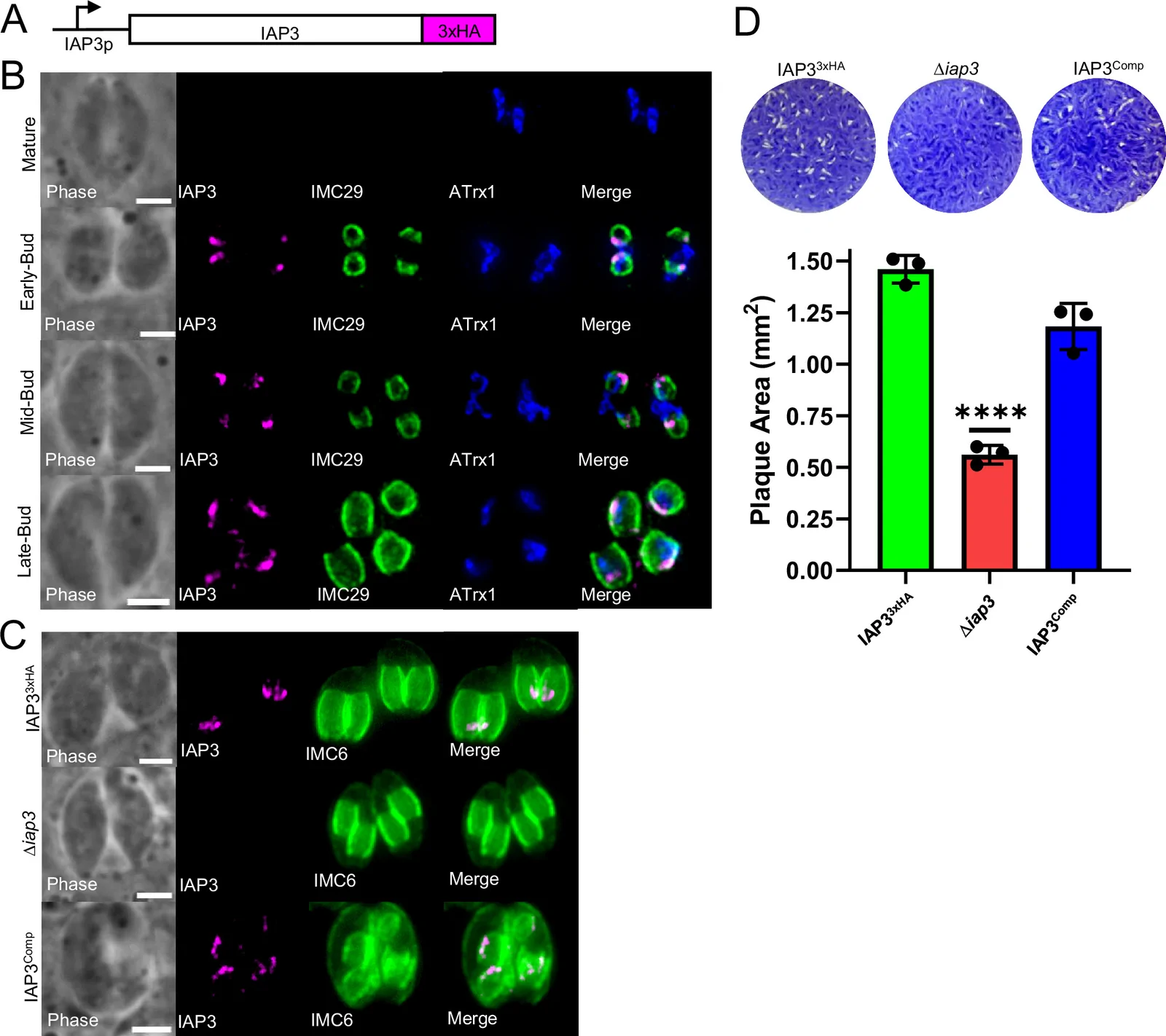
IAP3 is an IMC and apicoplast localizing daughter bud protein whose loss results in growth defects. **A** Diagram of IAP3 with a 3xHA C-terminal tag and no identifiable domains. **B** IFA time-course of IAP3 expression and localization with the developing IMC and apicoplast. Magenta, rabbit anti-HA; green, rat anti-IMC29; blue, mouse anti-ATrx1. **C** IFA showing IAP3^3xHA^ (top), Δ*iap3* (middle), and IAP3^Comp^ (bottom) parasites where IAP3 IFA signal was ablated in Δ*iap3* parasites and restored in IAP3^Comp^ parasites. Magenta, rabbit anti-HA; green, rat anti-IMC6. **D** Representative plaque images and quantification of plaque areas from IAP3^3xHA^ (*n* = 148), Δ*iap3* (*n* = 136), and IAP3^Comp^ (*n* = 165) strains. Data represented as mean ± SD, One-way ANOVA where p-values were corrected for multiple comparisons with Holm-Sidak correction (**** *p* < 0.0001). Three independent experiments were performed. Bars = 2 μm. Source data are provided as a Source Data file.

We previously identified IMC-associated protein 3 (IAP3, TgGT1_238170) from proximity labeling studies using the early daughter protein IMC29 as bait^20^. IAP3 is a 1557 amino acid protein that lacks identifiable domains, has a unique localization to the inner face of the IMC between two developing daughter buds, and is not present in maternal parasites. IAP3 also partially localizes with the apicoplast, which prompted us to investigate its localization in relation to the apicoplast and developing daughter IMC throughout the stages of endodyogeny. To do this, IAP3 was endogenously tagged at the C-terminus with a 3xHA epitope tag (IAP3^3xHA^, Fig. 1A). IAP3^3xHA^ was then costained with the apicoplast marker ATrx1 and the early daughter bud IMC marker IMC29 and imaged in mature parasites as well as through progressive stages of replication (Fig. 1B). In mature parasites, neither IAP3 nor IMC29 signals were present, confirming that IAP3 is only expressed during replication. During early and mid-budding, IAP3 is present and localized to one side of IMC29, as well as to the poles of the segregating apicoplast. After apicoplast segregation in the late budding stage, IAP3 remains localized to one side of IMC29 and is adjacent to the apicoplast.

To assess the function of IAP3, we disrupted its gene using CRISPR/Cas9 (Supplementary Fig. 1A), which was confirmed by PCR and the lack of staining by immunofluorescence assay (IFA) (*∆iap3*, Fig. 1C and Supplementary Fig. 1B). We then complemented the ∆*iap3* strain with full-length *IAP3* containing a C-terminal 3xHA tag targeted to the UPRT locus, which restored localization to one side of replicating daughter buds (IAP3^comp^). To investigate growth defects from the loss of IAP3, we conducted plaque assays comparing IAP3^3xHA^, *∆iap3*, and the IAP3^comp^ strains (Fig. 1D). Quantification of the plaque assays showed a 61% reduction in plaque area of ∆*iap3* parasites relative to the IAP3^3xHA^ strain, which was rescued by complementation. Despite these growth defects, we were unable to identify morphological abnormalities of any organelles. In addition, we found that the *∆iap3* strain compensates, with plaque areas returning to wild-type (WT) over time, making delineation of the molecular function of IAP3 challenging. However, due to the unique localization of IAP3 to one side of the IMC and its partial apicoplast colocalization, we utilized IAP3 as a tool to investigate other proteins in the IMC/apicoplast niche which may be relevant to replication and organelle inheritance.

### IAP3 TurboID identifies proximal proteins in the IMC inner membrane niche

**Fig. 2:**
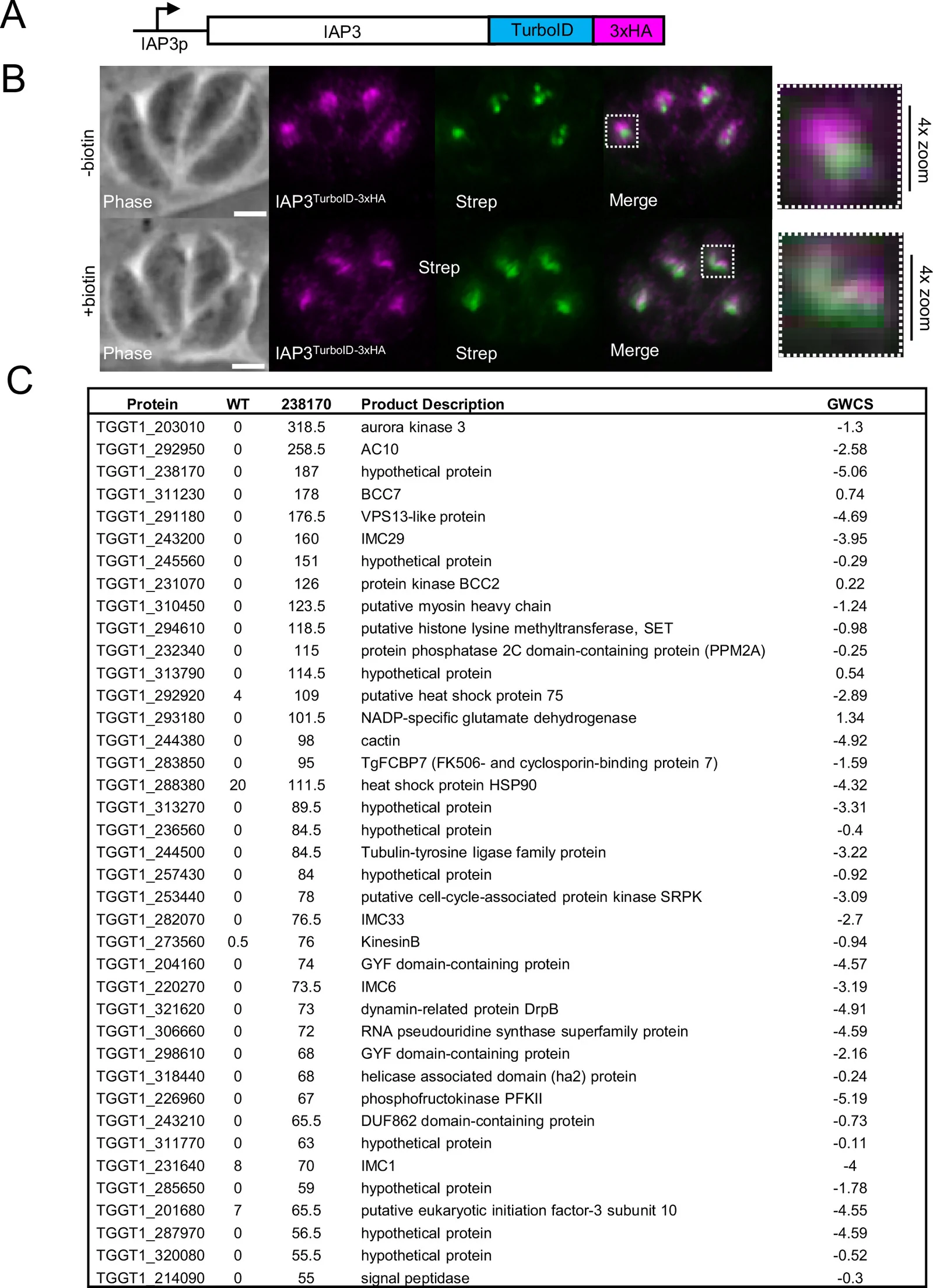
IAP3 TurboID identifies proximal proteins in the IMC inner membrane niche. **A** Diagram of the IAP3^TurboID-3xHA^ strain generated by fusing IAP3 with a C-terminal TurboID biotin ligase and 3xHA tag.** B** IFA images showing streptavidin (Strep) staining of the IAP3^TurboID-3xHA^ strain in (-) biotin (top) and (+) biotin (bottom) conditions. Streptavidin staining in (-) biotin controls detects endogenous biotinylation in the apicoplast adjacent to the bait. Dotted boxes (white) denote magnified regions, displayed on the right, showing increased streptavidin staining which colocalizes with IAP3^TurboID-3xHA^ after incubation with biotin. Magenta, mouse anti-HA; green, streptavidin-FITC. Bars = 2 μm. **C** Image showing the top 40 hits from proteomics analysis of untagged controls (WT) and IAP3^TurboID-3xHA^ showing spectral counts obtained. GWCS = the phenotype score from the *Toxoplasma gondii* genome wide CRISPR screen^21^. Three independent experiments were performed. Proteomics Source Data has been deposited in the massIVE platform under accession number MSV000101092.

To identify proteins that are involved with the nascent IMC and segregating apicoplast during endodyogeny, we endogenously tagged IAP3 with the TurboID biotin ligase plus a 3xHA tag for proximity labeling (IAP3^TurboID-3xHA^, Fig. 2A). To confirm the activity of the TurboID fusion, the IAP3^TurboID-3xHA^ line was incubated with or without biotin, then stained with streptavidin and imaged by IFA (Fig. 2B). The control group of IAP3^TurboID-3xHA^ parasites that were not incubated with biotin produced the expected background streptavidin staining from the endogenous biotinylation of the apicoplast. After incubation of IAP3^TurboID-3xHA^ parasites with biotin for 24 h, the streptavidin and IAP3^TurboID-3xHA^ staining overlapped, indicating that the TurboID fusion was actively biotinylating interactors of the IAP3 bait. After verifying the activity of the biotin ligase, we incubated WT control parasites and the IAP3^TurboID-3xHA^ strain with biotin for 24 h. The biotinylated products were then pulled down via streptavidin chromatography and identified by mass spectrometry. The hits were ranked based on normalized spectral counts relative to WT controls (Fig. 2C and Supplementary Data 1). The ranked hits were also assessed based on their phenotype score from a genome-wide CRISPR screen (GWCS)^21^, and whether they were hypothetical proteins of unknown function or had been previously characterized. Based on these criteria, we selected the uncharacterized protein TgGT1_287970 as a novel target of interest and sought to investigate its localization and function.

### TgGT1_287970 (APP1) is expressed during endodyogeny and localizes near the IMC, apicoplast, and centrosome

**Fig. 3:**
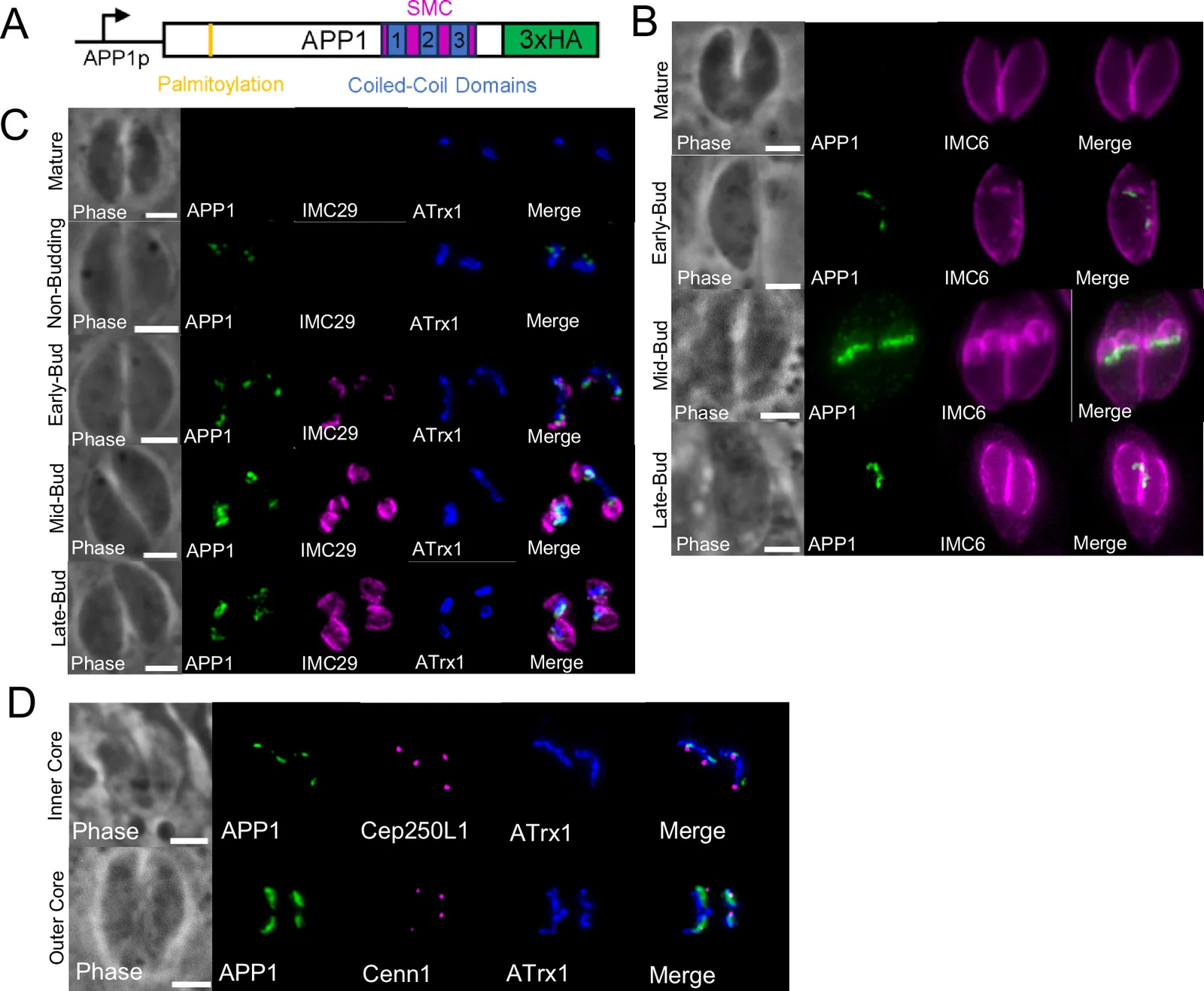
TgGT1_287970 (APP1) is expressed during endodyogeny and localizes near the IMC, apicoplast, and centrosome. **A** Diagram of APP1 featuring a predicted palmitoylation site (yellow) at residue 549, a structural maintenance of chromosomes (SMC) domain (pink) at residues 1421-1663, and 3 coiled-coil domains (blue) at residues 1545-1609, 1633-1659, and 1710-1763. **B** IFA images showing the localization of APP1 in relation to the maternal and daughter IMC marker IMC6. Green, rabbit anti-HA; Magenta, rat anti-IMC6. **C** IFA time-course of the development of APP1 with the early daughter bud marker IMC29 and the apicoplast marker ATrx1. Green, rabbit anti-HA; magenta, rat anti-IMC29; blue, mouse anti-ATrx1. **D** IFA images of the CEP250L1^Myc^ 287970^3xHA^ double tag strain (top) and 287970^3xHA^ tag strain (bottom) showing the localization of inner core centrosome marker CEP250L1 and outer core centrosome marker Centrin-1 in relation to 287970^3xHA^ and ATrx1. Magenta, rabbit anti-Myc (top); Magenta, rabbit anti-Centrin-1 (bottom); green, rat anti-HA; blue, mouse anti-ATrx1. Three independent experiments were performed. Bars = 2 μm.

TgGT1_287970 is a large protein of 2300 amino acids with a GWCS phenotype score of -4.59, a predicted palmitoylation site, and 3 predicted coiled-coil (CC) domains, which we named APP1 based on the results provided here^22–24^. The region containing the CC domains has a low similarity to a structural maintenance of chromosomes (SMC) domain, which is typically involved in DNA organization and segregation^25^. The protein appears to be restricted to the closest members of the family *Sarcocystidae* (e.g., present in *Hammondia spp* and *Neospora spp*, but absent in *Sarcosystis*). To localize APP1, the protein was endogenously tagged at the C-terminus with a 3xHA tag (APP1^3xHA^, Fig. 3A). APP1^3xHA^ was then assessed for its localization by IFA relative to the maternal and daughter IMC marker IMC6 (Fig. 3B). In mature parasites, APP1^3xHA^ signal was undetectable, suggesting a cyclic expression pattern that is only active during replication, akin to IAP3. During early and mid-budding, APP1^3xHA^ localized to the base of the daughter IMC, while in late budding stages, it localized to the inner face of the IMC daughter buds. This pattern was reminiscent of the segregating apicoplast, which prompted investigation of its expression relative to ATrx1 during replication (Fig. 3C). APP1^3xHA^ was again found to be absent in mature parasites but was localized to spots near the apicoplast prior to the expression of the early daughter marker IMC29. In the early and middle budding stages, APP1^3xHA^ localized to one side of the IMC of each daughter bud, as well as near the poles of the segregating apicoplast. In late budding stages, APP1^3xHA^ localized to one side of the daughter IMC and partially colocalized with the segregated apicoplast in each daughter bud. The simultaneous localization of APP1^3xHA^ to the daughter IMC and apicoplast was reminiscent of centrosomes, which are linked to the apicoplast and nucleus during replication to temporally coordinate their replication and inheritance into developing daughter buds^26,27^. Therefore, we colocalized APP1^3xHA^ with the centrosome inner core protein CEP250L1 and the outer core protein Centrin-1 (Fig. 3D). As expected, both CEP250L1 and Centrin-1 localized near the poles of the apicoplast. Although CEP250L1 was proximal to APP1^3xHA^, Centrin-1 appeared to be more closely colocalized with APP1^3xHA^, with APP1^3xHA^ being positioned between the apicoplast and Centrin-1. These studies suggest that APP1^3xHA^ may serve as a molecular adapter between the segregating apicoplast and the centrosome.

### Disruption of APP1 results in apicoplast and growth defects, but the centrosome is unaffected

**Fig. 4:**
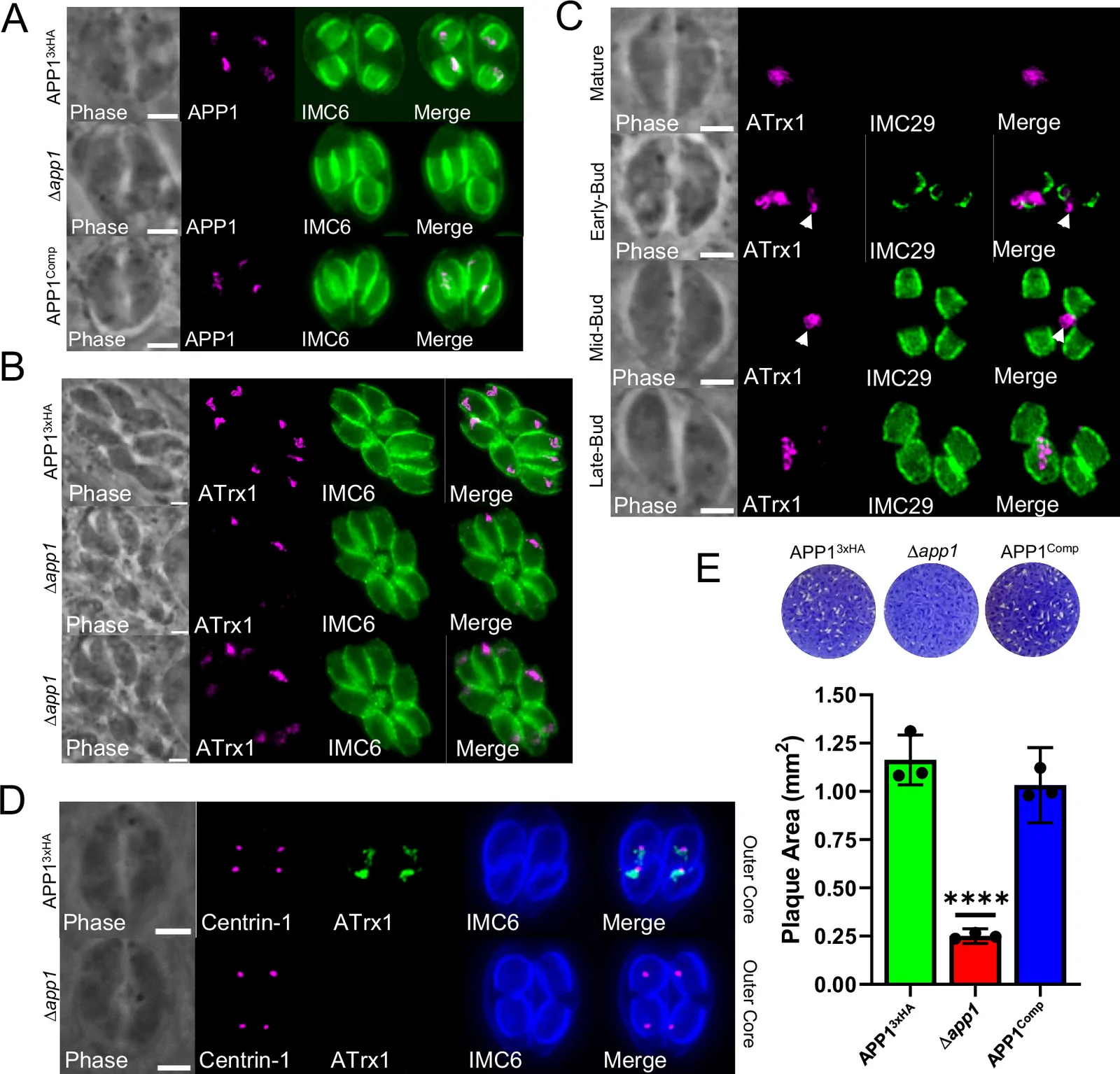
Disruption of APP1 results in apicoplast and growth defects, but the centrosome is unaffected. **A** IFA showing APP1^3xHA^ localizes with daughter buds labeled by IMC6, loss of staining in Δ*app1*, and restoration of localization in APP1^Comp^. Magenta, rabbit anti-HA; green, rat anti-IMC6. **B** IFA images showing APP1^3xHA^ parasites with a single, segregated apicoplast in each parasite (top), Δ*app1* parasites that have lost the apicoplast in six out of eight parasites (middle), and the same Δ*app1* parasite vacuole with enhanced ATrx1 signal showing the presence of ATrx1 spots (bottom). Magenta, mouse anti-ATrx1; green, rat anti-IMC6. **C** IFA images showing the localization of the apicoplast in relation to the early daughter bud protein IMC29 in Δ*app1* parasites. Arrowheads (white) indicate examples of apicoplast mislocalization from daughter buds. Magenta, mouse anti-ATrx1; green, rat anti-IMC29. **D** IFA of APP1^3xHA^ (top) showing partial colocalization of the apicoplast and Centrin-1, as well as localization of Centrin-1 to the daughter IMC. For the Δ*app1* line (bottom), ATrx1 signal is ablated but Centrin-1 staining and localization to daughter IMC is retained. Magenta, rabbit anti-Centrin-1; green, mouse anti-ATrx1; blue, rat anti-IMC6. **E** Plaque assays comparing plaque areas of APP1^3xHA^ (*n* = 150), Δ*app1* (*n* = 150), and APP1^Comp^ (*n* = 150) strains where average plaque areas were reduced by 78% for Δ*app1* parasites relative to APP1^3xHA^ plaque areas. Data represented as mean ± SD, One-way ANOVA where p-values were corrected for multiple comparisons with Holm-Sidak correction (**** *p *< 0.0001). Three independent experiments were performed. Bars = 2 μm. Source data are provided as a Source Data file.

To determine the function of APP1, we disrupted its gene using CRISPR/Cas9 (*∆app1*, Supplementary Fig. 2A). The resulting knockout strain lacked staining by IFA and was verified by PCR (Fig. 4A and Supplementary Fig. 2B). We then complemented the knockout with a full-length copy of APP1 driven from its endogenous promoter and targeted to the UPRT locus, which restored the staining pattern observed in the endogenously tagged line (APP1^comp^). Western blot analysis confirmed an absence of the protein in the knockout and that complementation resulted in rescue of the correctly migrating band, but at lower levels than that seen in the endogenously tagged strain (Supplementary Fig. 2C). We then assessed whether there were apicoplast defects present in the *∆app1* line by IFA (Fig. 4B). The APP1^3xHA^ line featured a singular apicoplast present in each parasite, as expected. However, the knockout showed parasites within vacuoles lacking apicoplasts, suggesting a failure of apicoplast partitioning. Longer exposures revealed small spots of ATrx1 at the usual site of the apicoplast. No morphological defects were identified in other organelles by IFA, indicating that *∆app1* parasites had apicoplast specific defects (Supplementary Fig. 3A, B).

To further investigate the apicoplast defect, we stained *∆app1* parasites with ATrx1 and IMC29 antibodies and imaged mature and budding parasites throughout endodyogeny (Fig. 4C). In addition to apicoplast loss, we noticed mislocalization of the apicoplast from the developing IMC, indicating a defect in apicoplast tethering to the developing daughter buds. These apicoplast inheritance abnormalities suggested a defect in association of the apicoplast with the centrosome, so we evaluated the localization of Centrin-1 and the apicoplast in the APP1^3xHA^ and Δ*app1* strains by IFA (Fig. 4D). As expected, the apicoplast partially colocalized with Centrin-1 in the APP1^3xHA^ line. In Δ*app1* parasites, apicoplast defects were present, but Centrin-1 was correctly localized to the daughter IMC, indicating that the loss of the apicoplast was not related to abnormalities in the localization or duplication of the centrosome. To quantify the effects of the loss of APP1 on parasite growth, we conducted plaque assays to compare the plaque areas of the APP1^3xHA^, Δ*app1*, and APP1^comp^ strains (Fig. 4E). We found a 78% reduction in plaque areas of the Δ*app1* line relative to APP1^3xHA^, which was rescued in APP1^comp^ parasites. The growth defects we observed in the plaque assay agree with the loss of the essential apicoplast. To determine if the mistiming or overexpression of APP1 could result in apicoplast inheritance defects, we complemented *∆app1* with a full-length complement that was driven by the strong and constitutively expressed tubulin promoter (APP1^pTubulin^, Supplementary Fig. 4)^28^. The APP1^pTubulin^ strain rescued apicoplast inheritance akin to the APP1^comp^ strain but resulted in diffusely cytoplasmic expression in mature parasites, indicating that constitutive over-expression alters the cell-cycle controlled expression of APP1 but does not significantly affect its function.

### Ultrastructure of wild-type, *∆app1*, and APP1^comp^ parasites

**Fig. 5:**
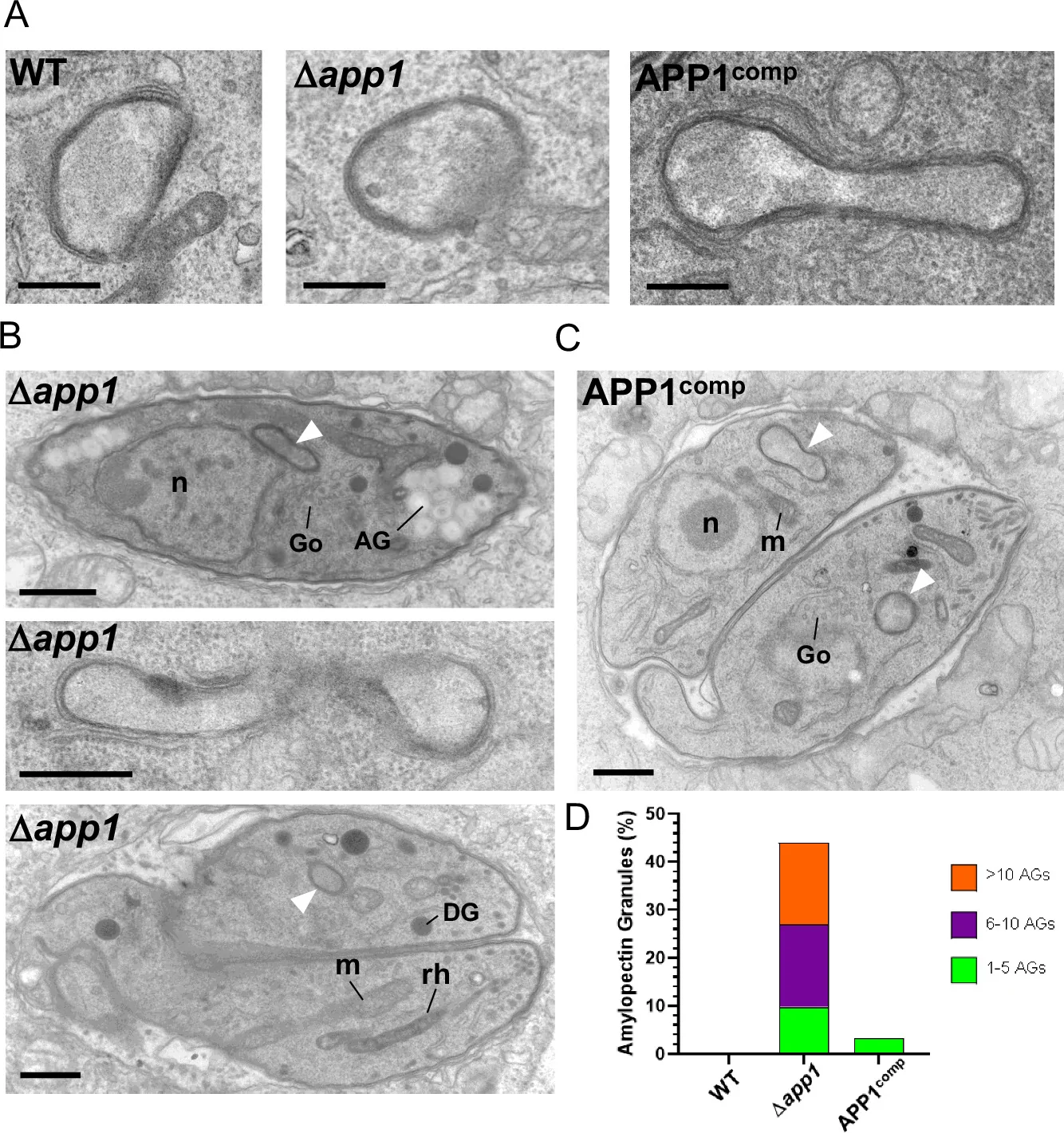
Ultrastructure of wild-type, *∆app1*, and APP1^comp^ parasites. **A**–**C** Electron microscopy of Δ*app1* parasites 24 h post-infection in HeLa cells. Representative images from 29 to 52 parasitophorous vacuoles are shown for the 3 experimental conditions. **A** Comparison of the ultrastructure of the apicoplast in WT (left), Δ*app1* (middle), and APP1^comp^ (right) parasites. **B** Δ*app1* parasites showing amylopectin granules (AG, top), elongated apicoplast (middle), and endodyogeny (bottom). **C** Complemented parasites showing healthy endodyogeny and lack of AGs. **D** Quantification of the percentage of WT, Δ*app1*, and APP1^comp^ parasites with AGs. WT parasites (*n* = 30) lacked any AGs, while ~3% of APP1^comp^ parasites (*n* = 29) had 1-5 AGs. For Δ*app1* parasites (*n* = 41), *~*44% of parasites had AGs and were sub-divided as follows: 9.8% had 1–5 AGs, 17.1% had 6–10 AGs, and 17.1% had >10 AGs per parasite. The legend corresponds to the number of AGs per parasite: Green (1–5), purple (6–10), and orange ( > 10). DG dense granule; Go Golgi; m mitochondrion; n nucleus; rh rhoptry. White arrowheads point to the apicoplast. Scale bars in **A**, 250 nm; **B** and **C**, 500 nm. Source data are provided as a Source Data file.

To corroborate our findings from IFA studies, we analyzed sections of WT, *∆app1*, and APP1^comp^ parasites by transmission electron microscopy (TEM, Fig. 5A–C). No differences were observed in the four-membrane morphology or size of the apicoplast between WT, ∆*app1*, and complemented parasites (Fig. 5A). While the WT parasites frequently showed large apicoplasts in apical sections, few apicoplastic profiles were visible in ∆*app1* parasites, which confirmed the loss of the apicoplast. ~44% of the knockout parasites contained amylopectin granules, a sign of stress during metabolic changes in tachyzoites, regardless of the presence or absence of an apicoplast (Fig. 5D)^29^. This is likely due to mitochondrial dysfunction due to the loss of metabolic products synthesized in the apicoplast that are utilized in the mitochondrion^30^. No other cytopathies were observed in other organelles, global endodyogeny, or the parasitophorous vacuole membrane ultrastructure in the ∆*app1* strain. At the onset of endodyogeny, the apicoplast elongated with a dumbbell intermediate shape in ∆*app1* parasites like in the WT strain, prior to scission. In complemented parasites, the appearance of the apicoplast was restored and the amylopectin granules were no longer present (Fig. 5C). Together with our IFA results, these data indicate that the loss of the apicoplast in ∆*app1* parasites is due to segregation of the organelle rather than gross morphological or membrane alterations that affect organelle partitioning.

### The APP1 C-terminus and residues 800-1408 are critical for apicoplast inheritance

**Fig. 6:**
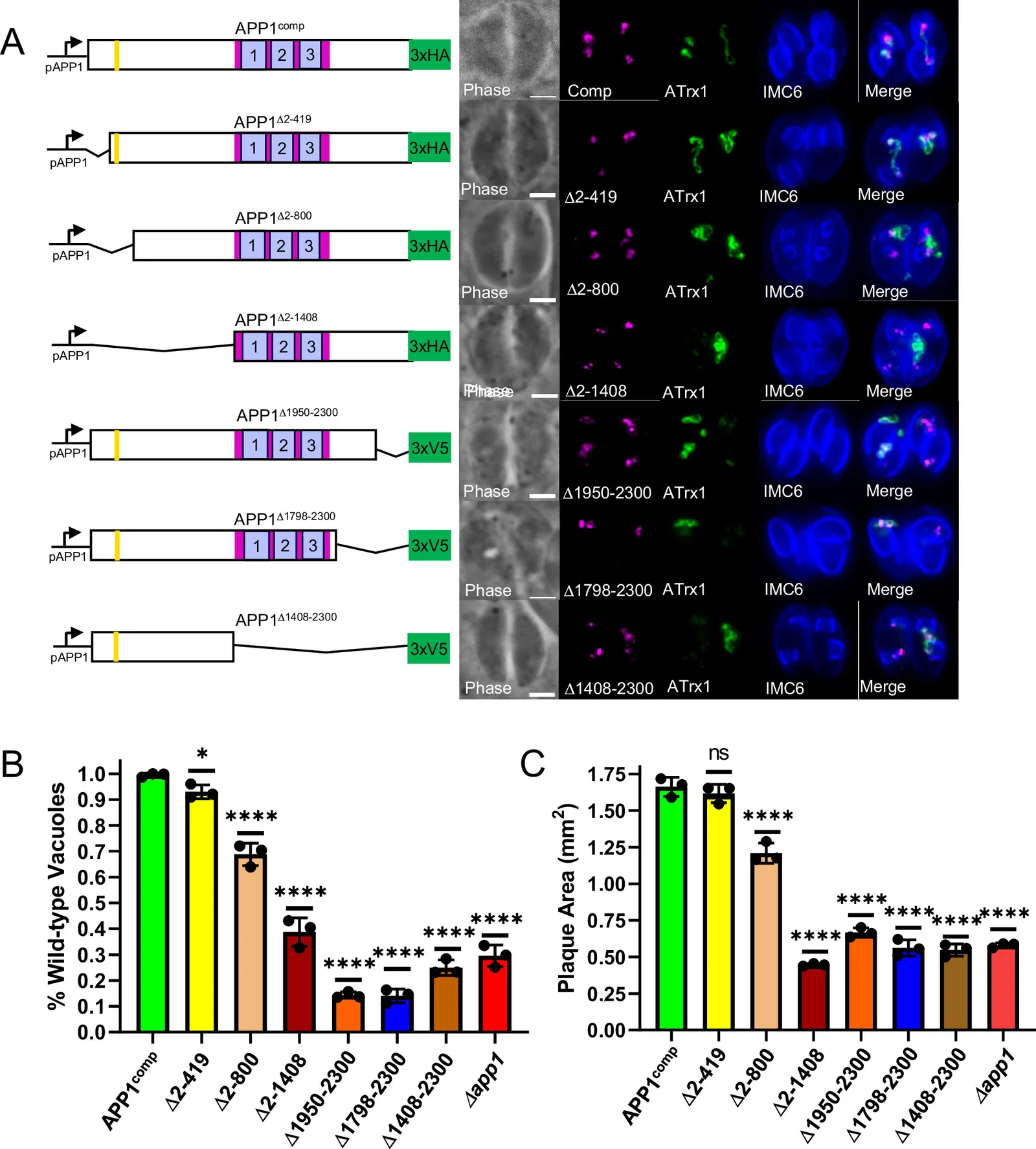
The APP1 C-terminus and residues 800-1408 are critical for apicoplast inheritance. **A** Diagrams of the APP1^comp^ deletion series with IFA images showing localization of the APP1 truncations with IMC6 and ATrx1. Magenta, rabbit anti-HA; green, mouse anti-ATrx1; blue, rat anti-IMC6. **B** Quantification of the percentage of wild-type vacuoles, with a singular apicoplast per parasite, for APP1^comp^ (*n* = 225), APP1^Δ2-419^ (*n* = 249), APP1^Δ2-800^ (*n* = 239), APP1^Δ2-1408^ (*n* = 238), APP1^Δ1950-2300^ (*n* = 249), APP1^Δ1798-2300^ (*n *= 245), APP1^Δ1408-2300^ (*n* = 233), and Δ*app1* (*n* = 225) strains. **C** Quantification of plaque areas between APP1^comp^ (*n *= 110), APP1^Δ2-419^ (*n* = 143), APP1^Δ2-800^ (*n* = 121), APP1^Δ2-1408^ (*n* = 150), APP1^Δ1950-2300^ (*n *= 138), APP1^Δ1798-2300^ (*n* = 99), APP1^Δ1408-2300^ (*n* = 122), and Δ*app1* (*n* = 131) strains. Data represented as mean ± SD, One-way ANOVA where p-values were corrected for multiple comparisons with Holm-Sidak correction (* *p* < 0.05, **** *p* < 0.0001). Bars = 2 μm. Three independent experiments were performed. Source data are provided as a Source Data file.

Based on the intriguing apicoplast phenotype, we decided to identify the regions of APP1 that are critical for its targeting and function via deletion analyses. The APP1^comp^ construct was truncated from the N-terminus (APP1^*∆2-419*^, APP1^*∆2-800*^, APP1^*∆2-1408*^) and was complemented into the *∆app1* background at the *UPRT* locus, while the C-terminus (APP1^*∆1950-2300*^, APP1^*∆1798-2300*^, and APP1^*∆1408-2300*^) was truncated endogenously with CRISPR/Cas9. The N- and C-terminal truncations were then imaged using IFA to assess targeting (Fig. 6A). The APP1^comp^ and APP1^*∆2-419*^ strains showed the expected partial localization to the apicoplast, although there were occasional instances of mislocalization in the APP1^*∆2-419*^ strain. In contrast, in the APP1^*∆2-800*^ and APP1^*∆2-1408*^ strains the truncated protein could be observed to be separated from the apicoplast, with mislocalization occurring at a higher frequency in the APP1^*∆2-1408*^ strain versus APP1^*∆2-800*^. In the C-terminal deletions, while many apicoplast null parasites were identified, the successful localization of APP1 with the apicoplast was better preserved than in the N-terminal deletions. This suggests a role for the N-terminal region in apicoplast tethering. Surprisingly, while the deletions span the entire length of the protein, none of these truncations resulted in mislocalization of APP1 from the daughter IMC, indicating that there are multiple attachment points between APP1 and the daughter IMC. We then quantified the percentage of parasite vacuoles with WT apicoplast inheritance in which a single apicoplast was present in each parasite at 24 h post-infection (Fig. 6B). Loss of the N-terminal 419 residues had an ~10% reduction in WT vacuoles relative to the complemented strain, while removal of 800 residues from the N-terminus resulted in an ~30% reduction in WT vacuoles. Further deletion from the N-terminus, or any of the C-terminal deletions, resulted in greater than 60% reductions in WT vacuoles, which was comparable to the level of reduction seen in ∆*app1* parasites (Fig. 6B). We also correlated these results with growth defects by conducting plaque assays and found that growth defects for each of the constructs largely mirrored the loss of the apicoplast (Fig. 6C). The APP1^*∆2-1408*^, APP1^*∆1950-2300*^, APP1^*∆1798-2300*^, and APP1^*∆1408-2300*^ truncations resulted in plaque areas which were similar to the *∆app1* strain, highlighting amino acids 800-1408 and the C-terminal 350 residues as critical regions for APP1 function.

### APP1 TurboID reveals a novel protein TgGT1_313360 which partially localizes to the apicoplast and centrosomes and is important for apicoplast partitioning

**Fig. 7:**
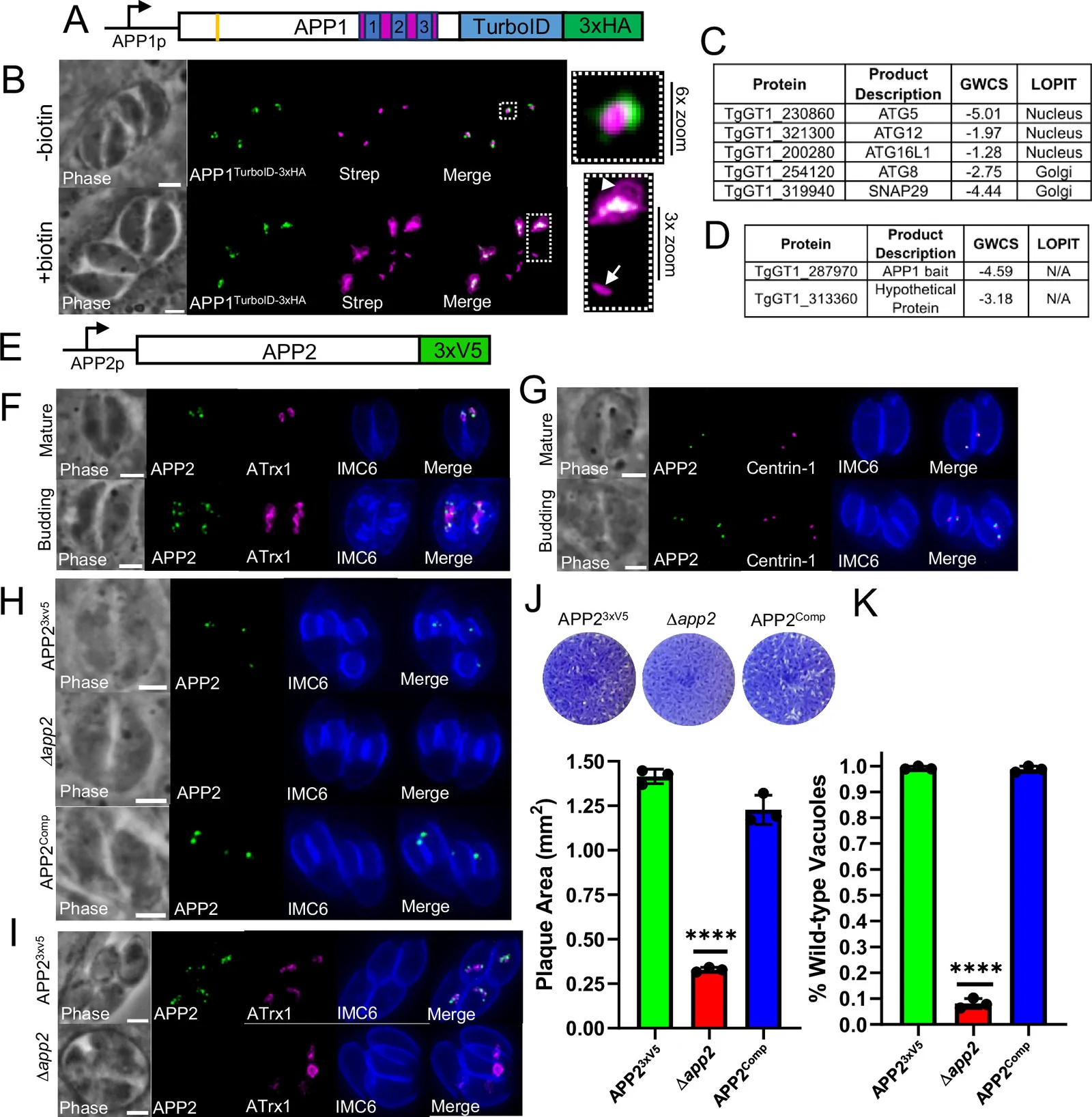
APP1 TurboID reveals a novel protein TgGT1_313360 which partially localizes to the apicoplast and centrosomes and is important for apicoplast partitioning. **A** Schematic of the APP1^TurboID-3xHA^ strain from C-terminal fusion of the TurboID biotin ligase and 3xHA epitope tag. **B** IFA images of the APP1^TurboID-3xHA^ strain without biotin (top) and with biotin (bottom). Dotted boxes (white) denote magnified regions, displayed on the right, showing the increased streptavidin staining colocalizing with APP1^TurboID-3xHA,^ the daughter IMC (arrowhead), and maternal basal complex (arrow). Green, mouse anti-HA; Magenta, rabbit streptavidin-FITC. **C** Table of previously characterized hits from the APP1 TurboID involved in apicoplast segregation. **D** Table comparing the APP1 TurboID bait and the uncharacterized hit TgGT1_313360 (APP2). **E** Schematic of 3xV5 epitope tagged APP2, which has no identifiable domains. **F** IFA images comparing APP2 and ATrx1 staining in mature (top) and budding parasites (bottom). APP2 forms punctate dots on the apicoplast and accumulates at the poles. Green, rabbit anti-V5; Magenta, mouse anti-ATrx1; blue, rat anti-IMC6. **G** IFA images comparing APP2 and Centrin-1 staining in mature (top) and budding parasites (bottom). APP2 partially localizes to Centrin-1 and is present in mature and budding parasites. Green, mouse anti-V5; Magenta, rabbit anti-Centrin-1; blue, rat anti-IMC6. **H** IFA showing APP2^3xV5^ expression, loss of staining in Δ*app2*, and restoration of expression in APP2^Comp^ parasites. Green, mouse anti-V5; blue, rat anti-IMC6. **I** IFA images of the APP2^3xV5^ tag strain showing APP2^3xV5^ expression and wild-type apicoplasts (top) and the Δ*app2* strain showing the loss of APP2 and apicoplasts from parasite vacuoles (bottom). **J** Plaque assays comparing plaque areas of APP2^3xV5^ (*n* = 153), Δ*app2* (*n* = 161), and APP2^Comp^ (*n* = 157) strains where average plaque areas were reduced by 77% for Δ*app2* parasites relative to APP2^3xV5^ plaques. **K** Quantification of percentage of wild-type vacuoles of APP2^3xV5^ (*n* = 239), Δ*app2* (*n* = 231), and APP2^Comp^ (*n* = 238) strains where Δ*app2* had a 92% reduction in wild-type vacuoles relative to APP2^3xV5^. Data represented as mean ± SD, One-way ANOVA where p-values were corrected for multiple comparisons with Holm-Sidak correction (****, *p* < 0.0001). Three independent experiments were performed. Bars = 2 μm. Source data are provided as a Source Data file.

To extend our investigation of the dynamics of apicoplast segregation, we endogenously tagged *APP1* at the C-terminus with a TurboID^-3xHA^ tag for proximity labeling studies (APP1^TurboID-3xHA^, Fig. 7A). The fusion protein targeted correctly, and the minus biotin controls showed the expected background in the apicoplast (Fig. 7B). The addition of biotin showed streptavidin staining overlapping with the fusion protein, and notably, faint staining could also be detected at the daughter IMC and basal complex. After verifying APP1^TurboID-3xHA^ activity, we incubated WT and APP1^TurboID-3xHA^ parasites with biotin for 24 h and conducted a streptavidin pulldown to capture biotinylated proteins for proteomics analyses (Supplementary Data 2). We then searched the ranked fold-change results by evaluating hits surrounding the APP1^TurboID-3xHA^ bait protein (log_2_ fold change relative to WT: 6.99) and noted the enrichment of proteins that were previously shown to participate in apicoplast inheritance (Fig. 7C)^16^. To identify novel uncharacterized proteins that could be involved in apicoplast inheritance, we screened for hits with CRISPR scores more negative than -3 and that lacked LOPIT localization data. Based on these criteria, we identified a hypothetical protein, TgGT1_313360 (log_2_ 7.93), as an interesting target for further study (Fig. 7D).

TgGT1_313360 encodes a 1485 amino acid protein with no identifiable domains and a GWCS score of -3.18 which we named APP2^21^. Similar to APP1, APP2 appears to be restricted to close relatives in the *Sarcocystidae*. To determine its localization, we endogenously tagged the C-terminus with the 3xV5 epitope tag (APP2^3xV5^, Fig. 7E). IFA staining of APP2^3xV5^ with ATrx1 showed that in mature parasites, APP2^3xV5^ formed a punctate dot near the apicoplast, while in budding parasites, it coated the dividing apicoplast and accumulated at the poles (Fig. 7F). To assess localization with the centrosome, we costained APP2^3xV5^ with Centrin-1 and found that the protein localized closely to the centrosome in both mature and budding parasites (Fig. 7G). These localization patterns prompted further investigation into whether APP2 is involved in apicoplast segregation.

Similarly to APP1, we disrupted APP2^3xV5^ via CRISPR/Cas9 (*∆app2*, Supplementary Fig. 5A) and complemented a second copy of the gene with a 3xV5 epitope at the *UPRT* locus (APP2^Comp^, Fig. 7H). IFA staining showed loss of 3xV5 signal in the knockout which was rescued by complementation. The knockout and complemented lines were confirmed by PCR and western blot analysis (Supplementary Fig. 5B, C). We then assessed whether disruption of APP2^3xV5^ resulted in apicoplast defects (Fig. 7I). Akin to ∆*app1*, the *∆app2* parasites frequently lost the apicoplast, and no morphological defects were identified in other organelles besides the apicoplast by IFA (Supplementary Fig. 6A, B). To quantify growth defects associated with loss of APP2, we conducted a plaque assay and found a 77% reduction in plaque area of the ∆*app2* strain relative to the APP2^3xV5^ strain, which was rescued in APP2^comp^ parasites (Fig. 7J). Quantification of WT vacuoles agreed with the plaque assay, where there was a 92% reduction in WT vacuoles in the ∆*app2* strain relative to the APP2^3xV5^ strain, which was also rescued in APP2^comp^ parasites (Fig. 7K). This data showed that loss of APP2 results in a defect in apicoplast partitioning similar to that seen in *∆app1* parasites and reveals a second player involved in apicoplast segregation.

### APP1 and APP2 localize closely but APP1 is IMC-associated while APP2 is more closely tethered to the apicoplast

**Fig. 8:**
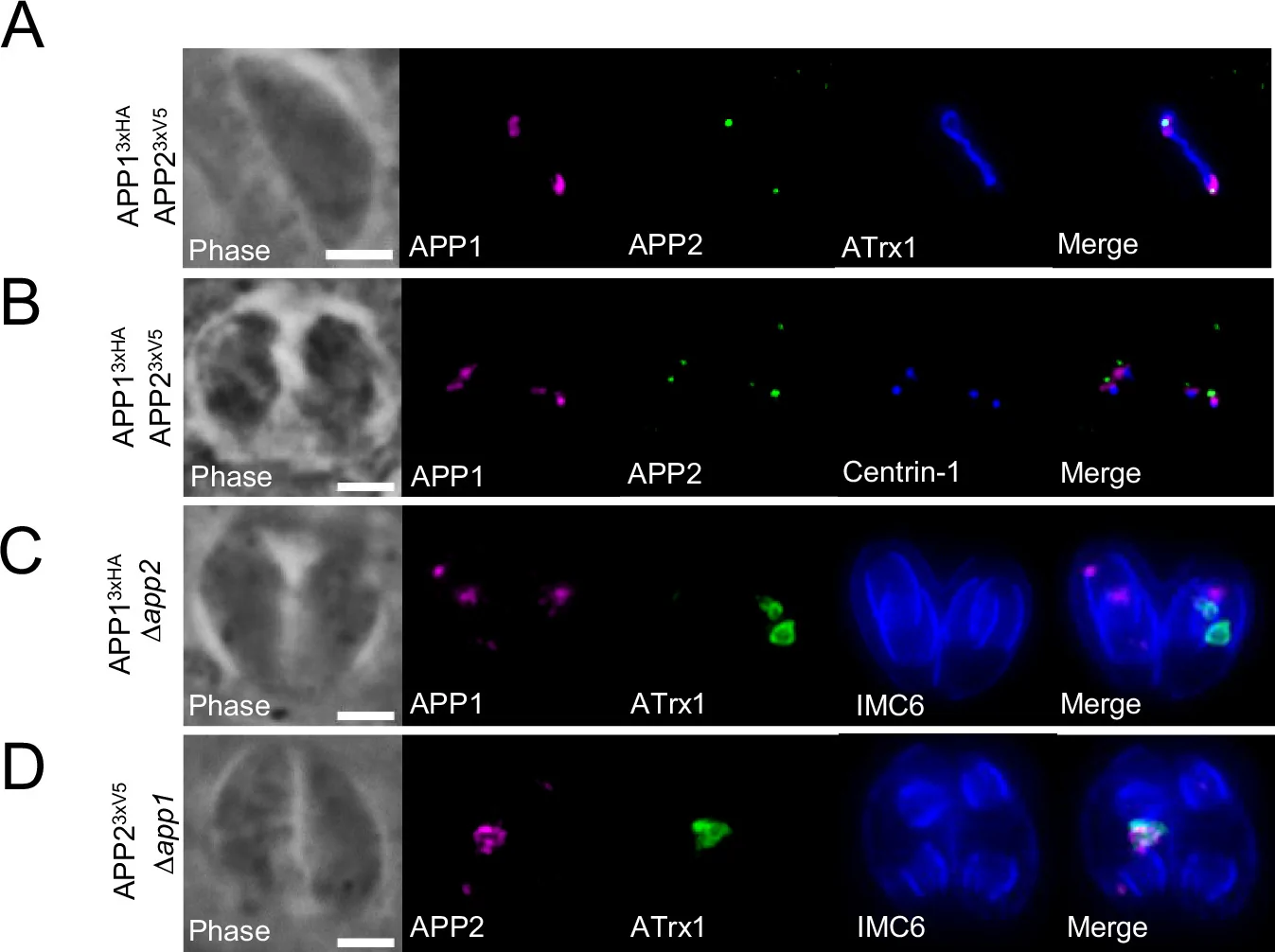
APP1 and APP2 localize closely but APP1 is IMC-associated while APP2 is more closely tethered to the apicoplast. **A** IFA demonstrating the partial localization of APP1 and APP2 at the poles of the segregating apicoplast. Magenta, rabbit anti-HA; green, rat anti-V5; blue, mouse anti-ATrx1. **B** IFA showing the localization of APP1, APP2, and Centrin-1. Magenta, mouse anti-HA; green, rat anti-V5; blue, rabbit anti-Centrin-1. **C** IFA showing the localization of APP1^3xHA^ in the Δ*app2* background where APP1^3xHA^ signal is concentrated on the daughter IMC. Magenta, rabbit anti-HA; green, mouse anti-ATrx1; blue, rat anti-IMC6. **D** IFA showing the localization of APP2^3xV5^ in the Δ*app1* background where APP2^3xV5^ signal is concentrated on the apicoplast. Magenta, rabbit anti-V5; green, mouse anti-ATrx1; blue, rat anti-IMC6. Three independent experiments were performed. Bars = 2 μm.

We decided to investigate the relative positioning of APP1 and APP2 to ascertain their relationships with each other and the apicoplast inheritance process. We tagged APP1 with 3xHA in the APP2^3xV5^ background and stained the APP1^3xHA^ APP2^3xV5^ strain with ATrx1. APP1 and APP2 aligned closely, but did not perfectly overlap, at the poles of the segregating apicoplast (Fig. 8A). We then examined the relative positioning of APP1, APP2, and Centrin-1; APP1 appeared to be positioned between Centrin-1 and APP2, suggesting that APP1 was more proximal to the cytoskeleton while APP2 was more proximal to the segregating apicoplast (Fig. 8B). To explore this hypothesis and the potential interdependence of APP1 and APP2, we tagged APP1^3xHA^ in the ∆*app2* background and tagged APP2^3xV5^ in the ∆*app1* background and assessed their localizations relative to the apicoplast and daughter IMC in budding parasites. When APP1^3xHA^ was tagged in the ∆*app2* background, the apicoplast was missegregated or missing due to the ∆*app2* apicoplast phenotype, and APP1^3xHA^ was localized to the daughter IMC and mislocalized from the apicoplast (Fig. 8C). When APP2^3xV5^ was tagged in the ∆*app1* background, the apicoplast was missegregated or missing due to the ∆*app1* apicoplast phenotype, and APP2^3xV5^ was highly enriched on the surface of the apicoplast but largely lost its expected localization to the daughter IMC (Fig. 8D). These findings demonstrate that in the context of the knockout of the other protein, APP1 is IMC-associated while APP2 is enriched on the apicoplast.

## Discussion

Our study has identified two novel proteins, APP1 and APP2, that localize near the centrosome, daughter IMC, and apicoplast and are involved in apicoplast inheritance in *T. gondii*. The cyclical expression and localization patterns of APP1 suggest that it functions as an adapter for the association of the dividing apicoplast with the centrosome, which we corroborated by finding apicoplast inheritance defects in the ∆*app1* strain (Fig. 4). When apicoplasts are present in the ∆*app1* strain, they are frequently mislocalized from the daughter IMC, which indicates a loss of association between the apicoplast and centrosome. This mislocalization is not attributable to a defect in the centrosomes as the centrosomes are properly duplicated and localized to the daughter IMC. TEM imaging of ∆*app1* parasites confirmed that the apicoplasts that are present contain a typical four-membrane bound, intact morphology (Fig. 5). In addition, amylopectin granules were frequently present in *∆app1* parasites whether or not an apicoplast was present, suggesting that the knockout is undergoing metabolic stress^31^. While the association between the apicoplast and the centrosomes is critical for the successful inheritance of the apicoplast, previous studies have shown that this association is not required for apicoplast elongation^13^. This agrees with our TEM data of the knockout, which showed that when present, the organelle is capable of undergoing elongation. Interestingly, small spots of ATrx1 are visible by IFA in longer exposures of the apical region of apicoplast null parasites in the ∆*app1* strain. As ATrx1 is nuclear encoded, these spots may be vesicles containing newly synthesized material that lacks an organelle to be trafficked to. However, no obvious structures corresponding to these vesicles were visible by TEM, although detection of these vesicles may be challenging. Together, our TEM and IFA analyses indicate that the defect is apicoplast specific, and inheritance appears to be related to segregation rather than organelle stability.

While APP1 lacks apparent functional domains aside from its SMC domain, our deletion analyses revealed important features of the protein (Fig. 6). APP1^∆2-419^ had minimal impact on the association of the apicoplast and daughter IMC. Further deletion in the APP1^∆2-800^ strain resulted in occasional mislocalization of the apicoplast, suggesting the presence of regions responsible for apicoplast tethering. A predicted palmitoylation site is present at residue 549 within this region, but this may not be authentic as it is not conserved in *Neospora caninum*. Deletion up to the SMC domain in APP1^∆2-1408^ parasites results in severe apicoplast partitioning defects comparable to the *∆app1* strain, indicating that residues 800-1408 are important for linkage to the apicoplast. All of the C-terminal truncations recapitulated the defects seen in the *∆app1* strain, highlighting the C-terminal 350 amino acids as critical for function. The large deletions used here may affect protein folding, but all of the truncations were able to successfully recruit to the daughter IMC. This argues against gross folding or structural defects and suggests that APP1 contains multiple attachment points to the inner face of the daughter IMC. Our APP1 proximity labeling experiments contain several daughter bud enriched alveolins (e.g., IMC3, IMC6, and IMC10) which would be in close proximity to the apicoplast and good candidates for daughter IMC tethering. IMC10 has previously been shown to link the mitochondrion to the IMC, demonstrating that alveolins play a role in organelle positioning^32^. However, knockdown of IMC10 did not appear to affect the apicoplast. Similarly, we previously disrupted IMC6, which resulted in dramatic morphological abnormalities but no apicoplast defects^33^. IMC3 represents a potential candidate for the cytoskeletal association of APP1 due to its cytoskeletal domains and expression timing that corresponds to apicoplast elongation^34^. It is also possible that APP1 is tethered to the daughter IMC via more than one alveolin or alveolin associated protein, which may only be revealed upon the disruption of multiple partners.

Proximity labeling of APP1 as bait identified APP2, which localizes to discrete spots adjacent to APP1, as well as near the apicoplast, daughter IMC, and centrosome. APP2 can also be detected along the length of the elongating apicoplast, further suggesting a role in apicoplast inheritance, which was confirmed by disruption of *APP2* (Fig. 7). Unlike APP1, the constitutive expression of APP2 and its coating of the apicoplast with punctate dots resembles the expression and localization patterns of ATG8^16^. This suggests that APP2 may participate in the linkage of ATG8 on the apicoplast to the centrosome, as the apicoplast and centrosome are linked via ATG8 not only through replication stages but also during interphase^14^. Future studies examining ATG8 in the context of APP2 in WT and ∆*app2* parasites will help to validate this hypothesis. In addition, APP2 proximity labeling and co-immunoprecipitation studies will provide important leads on its interactors and clarify its relationship to ATG8.

Examination of APP1 and APP2 in the knockout strains provided insight into their links to the apicoplast and daughter IMC, with APP1 remaining associated with the forming daughter IMC in the ∆*app2* strain, and APP2 becoming largely relegated to the apicoplast in the ∆*app1* strain (Fig. 8). It is possible that the bridging of the apicoplast and daughter IMC is mediated by a direct interaction between APP1 and APP2, which will be further investigated via co-immunoprecipitation and pairwise yeast two-hybrid studies. Our studies are complemented by the co-submitted paper from Eichlin et al. who also discovered APP1 and its apicoplast inheritance defects using a conditional knockdown system. The conditional knockdown system utilized by Eichlin et al. corroborated our findings of apicoplast inheritance defects seen in our complete knockout. Using APP1 proximity labeling, they identified another factor, APP3, which also produces apicoplast defects on depletion. Interestingly, APP3 was not enriched in our biotinylation studies, while APP2 was poorly enriched in the Eichlin et al. study. This may be due to the fact that our studies used C-terminally TurboID tagged APP1 while theirs employed an N-terminally tagged version. The discovery of these factors by both of our groups demonstrates the complexity of the coordination of the connection of the apicoplast to the centrosome and IMC, and that there may be additional unknown factors involved.

Future studies will elucidate the precise mechanisms by which APP1 and APP2 facilitate the association between the elongating apicoplast and duplicated centrosomes. ATG8 conjugated to phosphatidylethanolamine (ATG8-PE) on the exterior apicoplast membrane has been shown to be involved in the linkage of the apicoplast to the centrosome^14^, but other factors involved in the linkage between ATG8 and the centrosome remain unknown. One possibility is that APP1 and APP2 interact with ATG8-PE to support centrosomal association. As ATG8 frequently interacts with its targets via AIMs/LIRs, we searched the sequences of APP1 and APP2 for AIMs/LIRs with iLIR, an AIM prediction software^15^. We did not identify motifs that were strongly predictive of an interaction between ATG8 and APP2. However, we found an xLIR motif on APP1 in an anchor position from residues 63-68 and a WxxL motif in an anchor position from residues 274-279^35,36^. However, the APP1^∆2-419^ strain had negligible effects on apicoplast-centrosome association or on parasite growth. Furthermore, ATG8 was poorly enriched in the APP1 TurboID (log_2_ 2.31). Therefore, ATG8 is less likely to be a direct binding partner of APP2 or APP1, although there may be interactions between APP1 and ATG8 outside of the extreme N-terminus of APP1.

Unlike ATG8, factors on peri-apicoplast vesicles recruited to the niche between the elongating apicoplast and developing daughter IMC were highly enriched in the APP1 TurboID. For example, ATG5 (TgGT1_230860, log_2_ 5.84), ATG12 (TgGT1_321300, log_2_ 6.76), ATG16L1 (TgGT1_200280, log_2_ 5.09), and SNAP29 (TgGT1_319940, log_2_ 8.62) were all highly enriched in the APP1 TurboID. The ATG5-12-16L1 complex is required for lipidation of ATG8 with PE, while SNAP29 is required for the fusion of peri-apicoplast vesicles onto the apicoplast outer membrane^16^. APP1 may therefore be involved in the coordination of the conjugation of ATG8 to PE, or vesicle recruitment and fusion events of peri-apicoplast vesicles, rather than direct interactions with ATG8. The production of apicoplast targeted vesicles is dramatically increased during apicoplast elongation, which mirrors the cell-cycle dependent expression of APP1. However, the presence of ATrx1 spots in the apical region of ∆*app1* parasites that are apicoplast null suggests that vesicle recruitment is not disrupted by the loss of the apicoplast. Another possibility is that APP1 is interfacing with the actomyosin system during apicoplast elongation. MyoF depletion leads to the accumulation of apicoplast protein vesicles in the cytoplasm, but MyoF depletion has minimal effects on vesicle motion, indicating that MyoF and actin are important for a different aspect of vesicle delivery such as vesicle fusion^13^. However, MyoF and actin were not well enriched in the APP1 TurboID. It is therefore possible that rather than being a direct binder of actin, APP1 may indirectly facilitate the organization of the actomyosin system around the apicoplast for vesicle fusion or the association of the actomyosin system to the centrosome.

Together, our study and that of Eichlin et al. identify APP1-3, a series of novel players that play key roles in apicoplast segregation. Further investigation of apicoplast vesicle fusion processes and the contributions of the actomyosin system to apicoplast inheritance will clarify how APP1-3 complete the missing link in apicoplast-centrosome association in *T. gondii*.

## Methods

### Toxoplasma and host cell culture

*T. gondii* RH*Δku80Δhxgprt* parasites and engineered strains were maintained on confluent monolayers of human foreskin fibroblast (HFF) host cells at 37 °C in a humidified atmosphere containing 5% CO₂. Parasites were cultured in Dulbecco’s Modified Eagle’s Medium (DMEM) supplemented with 5% fetal bovine serum (Gibco), 5%Cosmic Calf serum (HyClone), and penicillin-streptomycin-L-glutamine (Gibco). Endogenously tagged parasites with selectable markers were isolated by drug selection using 40 μM chloramphenicol (CAT), 1 μM pyrimethamine, or 50 μg/ml mycophenolic acid with 50 μg/ml xanthine (HXGPRT)^37–39^. Selection for homologous recombination at the *UPRT* locus was carried out using 5 μM 5-fluoro-2’-deoxyuridine (FUDR)^40^.

### Sequence analysis

*T. gondii* sequences were obtained from ToxoDBv68^41^ and placed in ApE v3.1.5. Sequence domains were searched with the NCBI conserved domain search (CD-search) tool, SMARTv10, and Interpro v108^42–44^. Palmitoylation sites were identified with CSS Palm 2.0, and the coiled-coil domains were identified with DeepCoil 2.0.2^22–24^.

### Chemicals and antibodies

Detection of hemagglutinin (HA) epitope was done with mouse monoclonal antibody (mAb) anti-HA.11 (1:1000, BioLegend, #901515, 16B12), rabbit mAb anti-HA (1:1000, Cell Signaling Technologies, #3724, C29F4), or rat mAb anti-HA (1:1000, ChromoTek, 7C9). The V5 epitope was detected with mouse mAb anti-V5 (1:1000, Invitrogen, R96025, SV5-Pk1), rabbit mAb anti-V5 (1:1000, Cell Signaling Technologies, #13202, D3H8Q), or rat mAb anti-V5 (1:1000, Abcam, #Ab206571, SV5-P-K). The c-Myc epitope was detected with mouse mAb anti-Myc (1:1000, Invitrogen, #MA1-980, 9E10) and rabbit polyclonal antibody (pAb) (1:1000, ProteinTech, #Ag9409, 16286-1-AP). Antibodies specific to *T. gondii* were rat anti-IMC29 (1:1000)^20^, rabbit pAb anti-IMC6 (1:1000)^34^, rat anti-IMC6 (1:1000)^45^, mouse mAb anti-ATrx1 (1:1000, 11G8)^46^, rabbit pAb Centrin-1 (1:1000, Kerafast, EBC004), mouse anti-ROP7 (1:1000)^47^, mouse anti-MIC2 (1:1000)^48^, mouse anti-F1β ATPase (1:1000)^49^, rat anti-DrpB (1:1000)^50^, guinea pig anti-NHE3 (1:1000)^51^, mouse anti-GRA14 (1:1000)^47^.

### Immunofluorescence assays and western blotting

HFF cells grown to confluence on glass coverslips were infected with *T. gondii* parasites expressing epitope tags. After 18-36 h of infection, coverslips were fixed with either 3.7% formaldehyde or 100% methanol and processed for IFA^52^. Primary antibodies were visualized using species-specific secondary antibodies conjugated to Alexa Fluor 594, 488, or 405. TurboID fusion constructs were validated by assessing functional biotinylation using Texas Red–conjugated streptavidin (Invitrogen, S872). Coverslips were mounted in Vectashield (Vector Laboratories) and examined using an Axio Imager.Z1 fluorescence microscope (Zeiss). Images were processed with ZEN Blue 3.7 software (Zeiss), including deconvolution and adjustment of the magenta pseudo-color corresponding to the 594 nm fluorophore. Image cropping was conducted in Photoshop 2026 v27.9.1. For western blot analysis, parasites were lysed in 1× Laemmli sample buffer (50 mM Tris-HCl [pH 6.8], 10% glycerol, 2% SDS, 100 mM DTT, 0.1% bromophenol blue) and boiled at 100 °C for 5 min. Lysates were separated by SDS–PAGE and transferred to nitrocellulose membranes. Membranes were incubated with the appropriate primary antibodies followed by horseradish peroxidase (HRP)-conjugated secondary antibodies. Proteins were detected by chemiluminescence (Thermo Scientific) and imaged using a ChemiDoc XRS+ system (Bio-Rad) with Image Lab 5.1.

### Epitope tagging and gene knockout

For endogenous C-terminal tagging, guides were generated 100 to 200 base pairs downstream of the stop codon of each gene, and were ligated into a pU6-universal plasmid^53^. LIC (ligation-independent cloning) tagging vectors with 40 base pair flanks were amplified from a homology-directed repair template with a selection cassette for recombination at the 3′ untranslated region (UTR) of each gene^54^. This template was PCR amplified, purified by phenol-chloroform extraction, and precipitated with ethanol. The purified DNA and 50 μg of pU6-universal guide were then electroporated into RHΔ*hxgprt*Δ*ku80* parasites. HFF cells were infected with transfected parasites, and drug selection based on the selectable marker was conducted (1 μm pyrimethamine, 50 μg/ml mycophenolic acid/xanthine, or 1 μm chloramphenicol). Parasites were screened by IFA, and clonal lines that had been successfully transfected were isolated via limiting dilution.

For gene knockouts, guides were generated to target an exon in each gene of interest. Homology-directed repair (HDR) templates including the selectable marker HXGPRT driven by the NcGRA7 promoter, 40 bp of homology immediately upstream of the start codon, and 40 bp of homology ~200 bp downstream of the stop codon were amplified for each gene. The NcGRA7 promoter was used to limit *T. gondii* sequences that might enable off-target recombination. For transfection, guides and HDR templates were purified as above and transfected into endogenously tagged parasite strains. The knockout strains were evaluated for loss of the tag by IFA and verified by PCR. All primers used for PCR verifications and the generation of tag, knockout, deletion, and complemented strains are denoted in Supplementary Data 3.

### Quantification of vacuoles with wild-type apicoplasts

Complemented and truncated strains of APP1 were seeded on confluent HFF coverslips and allowed to replicate for 24 h. Coverslips were fixed with 3.7% formaldehyde, blocked, and stained for ATrx1 and IMC6 for immunofluorescence quantification of vacuoles with WT and mutant apicoplasts. Fifty vacuoles ( > 4 parasites per vacuole) were counted for each replicate over three biological replicates. Vacuoles with a single apicoplast in each parasite were considered WT, while vacuoles with any parasite that was apicoplast null were considered as vacuoles with apicoplast defects. Data were represented as mean ± SD. Statistical significance was determined via one-way ANOVA, multiple comparisons test with Holm-Sidak correction. Graphs and figures were generated using Prism GraphPad 8.0.

### Plaque assays

Plaque assays were conducted in six-well plates with confluent HFF monolayers. Parasites were allowed to grow for 7 days, and then were fixed with cold methanol and stained with crystal violet for plaque visualization^55^. For each strain, three biological replicates with 50 plaques per replicate were imaged in ZEN Blue 3.7 software (Zeiss) and plaque areas were measured in ImageJ 2.16.0/1.54p. Data were represented as mean ± SD. Statistical significance was determined via one-way ANOVA, multiple comparisons test with Holm-Sidak correction. Graphs and figures were generated using Prism GraphPad 8.0.

### Complementation and deletion series generation

To generate the APP1 and APP2 full-length complement plasmids, the coding regions were PCR amplified from a cDNA library, and the endogenous promoters of each gene were PCR amplified from preparations of genomic DNA. Promoters, coding regions, and UPRT-locus knockout vectors were then annealed to produce the circularized complement plasmids with the NEBuilder HiFi DNA assembly master mix (NEB, E2621S)^56^. The online NEBuilder Assembly Tool v2.11.0 (https://nebuilder.neb.com/#!/) was used to design primers for generating complementation constructs. The N-term deletion constructs required for the deletion series of APP1 were generated by KLD mutagenesis (NEB, E0554) of the full-length complement strains, and the primers were designed using the online NEBaseChanger v2.8.4 (https://nebasechanger.neb.com). The plasmid constructs were linearized with PsiI-v2 (NEB), purified and precipitated by phenol-chloroform extraction and ethanol precipitation, and transfected into the knockout parasites along with a universal pU6 guided to UPRT. Selection for successful transfection and integration of linearized plasmids at the UPRT locus was conducted with FUDR^40^. APP1^comp^ and APP2^comp^ expressing clones were confirmed by IFA for 3xHA and 3xV5 epitopes, respectively. The N-terminal deletion constructs of APP1 were confirmed by IFA for the 3xHA epitope.

### Transmission electron microscopy

For ultrastructural observations of infected cells by thin section, samples were fixed in 2.5% glutaraldehyde in 0.1 mM sodium cacodylate and processed as described previously^57^. Ultrathin sections of infected cells were stained with osmium tetroxide before examination with Hitachi 7600 EM under 80 kV equipped with a dual AMT CCD camera system.

### TurboID proximity labeling

The proximity labeling experiments were conducted according to the detailed protocol we previously published^58^. The TurboID fusion proteins were generated according to the Epitope tagging and gene knockout section of methods. For large scale TurboID labeling, the fusion protein and an untagged WT strain were incubated in the presence of biotin for 24 h before streptavidin purification.

### MS sample preparation

Proteins bound to streptavidin beads were reduced with 5 mM TCEP and alkylated with 10 mM iodoacetamide by sequential 20-min incubations at room temperature in the dark, with shaking at 1200 rpm on an Eppendorf Thermomixer. Proteins were then digested overnight at 37 °C with 0.1 µg Lys-C (FUJIFILM Wako, 125-05061) and 0.8 µg Trypsin (Thermo Scientific, 90057). Digestion was quenched by adding formic acid to a final concentration of 5% (v/v). Peptides were desalted using C18 tips (Thermo Scientific, 87784), eluted, and resuspended in 15 µL of 5% formic acid prior to LC-MS/MS analysis.

### LC-MS/MS data acquisition and processing

For the 238170 dataset, peptides were separated on a 75 µm inner diameter × 25 cm C18 column (packed with 1.9 µm C18 resin, Dr. Maisch GmbH) over a 140-min gradient of increasing acetonitrile. Eluted peptides were analyzed on a Thermo Orbitrap Fusion Lumos mass spectrometer using data-dependent acquisition (DDA) mode. Database searching was performed using ProLuCID v3.1 with DTASelect2 v2.1.3 as implemented in the Integrated Proteomics Pipeline v5.0.1 (IP2; Integrated Proteomics Applications) against the *T. gondii* GT1 proteome downloaded from ToxoDBv68. Peptide-spectrum matches (PSMs) were filtered using a target–decoy database competition strategy to achieve a PSM-level false discovery rate (FDR) < 1%. Protein identifications were assembled and filtered in DTASelect2 v2.1.3, requiring a minimum of two unique peptides per protein. For the APP1 dataset, peptides were analyzed using a Thermo Scientific Vanquish Neo UHPLC system coupled to a Thermo Orbitrap Astral mass spectrometer via a nano-ESI source. Peptides were loaded onto a 150 µm × 15 cm C18 column and separated at 1.8 µL/min. Data were acquired in positive ion mode using data-independent acquisition (DIA). DIA data were processed using DIA-NN v1.9 in library-free direct DIA mode. In silico spectral libraries were generated from the *T. gondii* GT1 proteome of ToxoDBv68. Enzyme specificity was set to trypsin, with carbamidomethylation of cysteine as a fixed modification. DIA-NN v1.9 was run in double-pass mode with default quantification and neural-network scoring settings. Statistical analysis was performed using FragPipe Analyst v1.19.

## Supplementary information

Supplementary Information

Supplementary Dataset 1

Supplementary Dataset 2

Supplementary Dataset 3

Transparent Peer Review file

## Source data

Source data

## Data Availability

Source Data are provided with this paper. The Proteomics Source Data generated in this study has been deposited in the massIVE database under accession code MSV000101092 (https://doi.org/10.25345/C5PK07F9N). Source data are provided with this paper.
