# Supplementary material for: Identification and characterization of factors that govern apicoplast segregation in Toxoplasma gondii
Source: Nat Commun. 2026 Oct 5;17:10369. doi: 10.1038/s41467-026-78102-x (PMC13639046; doi:10.1038/s41467-026-78102-x)
Supplement: Supplementary file 1 [file 41467_2026_78102_MOESM1_ESM.pdf]

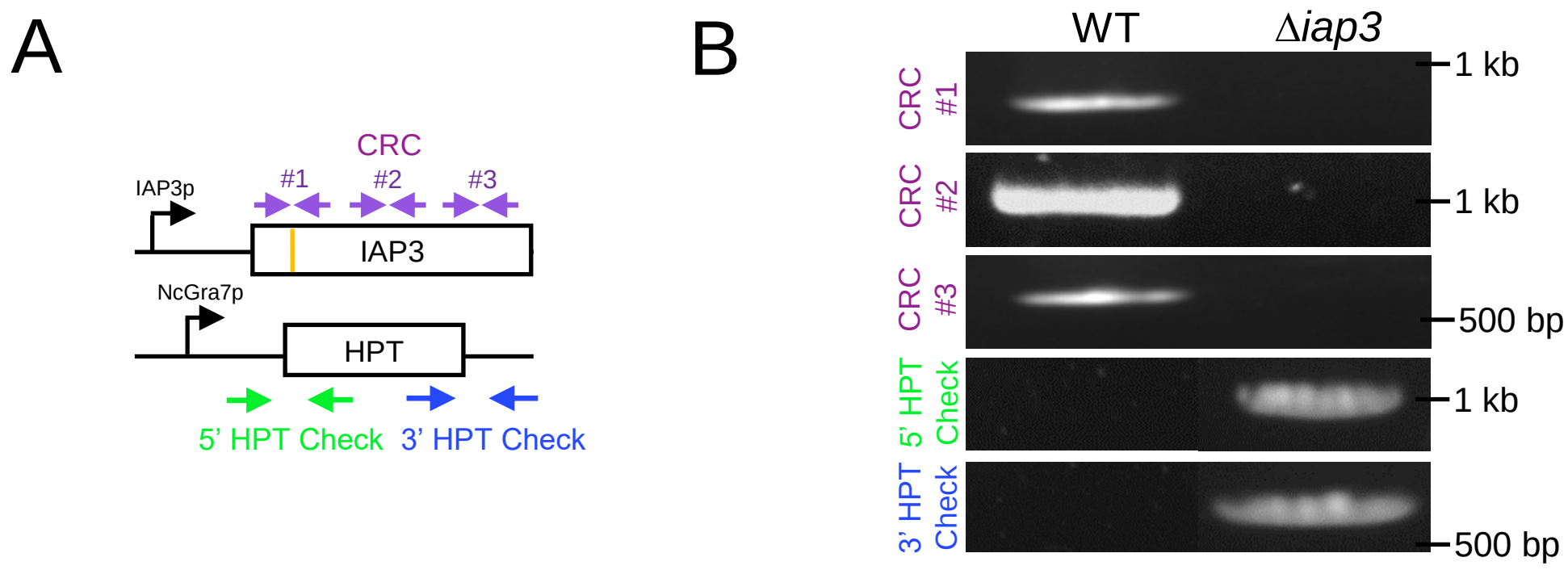

Supplementary Figure 1. PCR verification of Δ*iap3* parasite strain. A) Schematic of primer design for validation of the Δ*iap3* strain. The Cas9 guide sequence from base pairs 343-362 is indicated by an orange line. B) PCR verification of the Δ*iap3* strain at the gene of interest (GOI) and forward and reverse recombination checks after recombination of the HXGPRT (HPT) cassette. CRC, coding region check. Amplicons of CRC #1, #2, and #3 were 796 bp, 1013 bp, and 698 bp, respectively. Amplicons of 5' HPT Check and 3' HPT Check were 1012 bp and 605 bp, respectively. Molecular weight markers are denoted by kilobase (kb) or base pair (bp) markers.

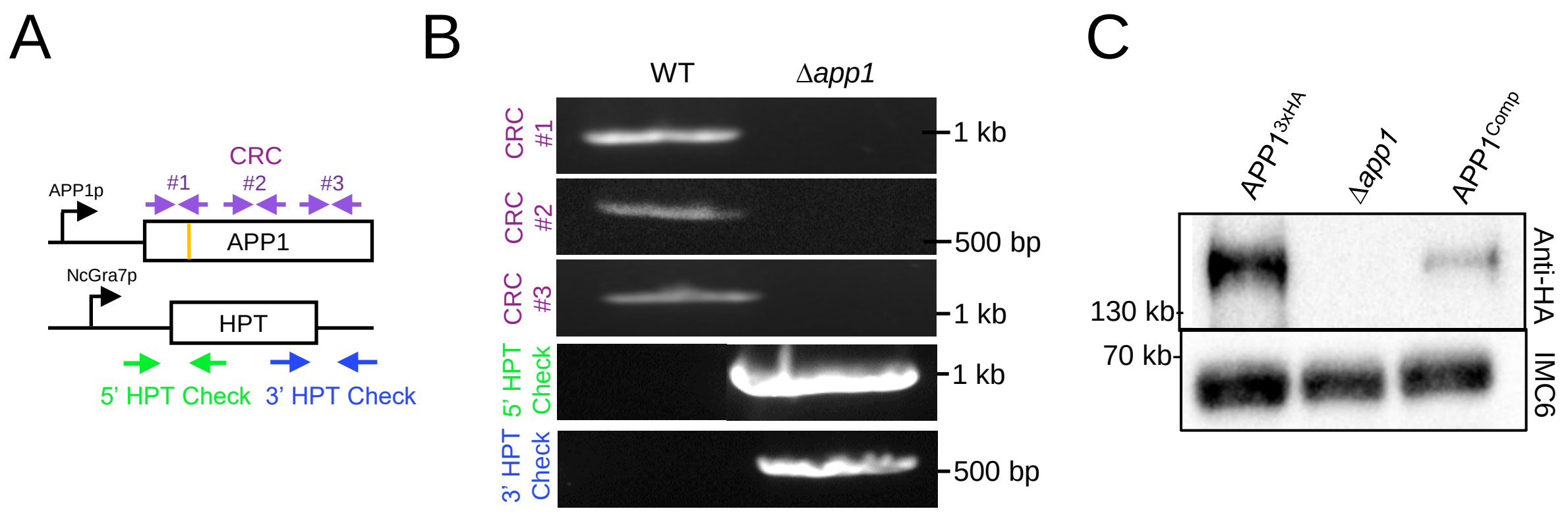

Supplementary Figure 2. PCR verification and western blot of knockout and complemented APP1 strains. A) Schematic of primer design for validation of the Δ*app1* strain. The Cas9 guide sequence from base pairs 1750-1769 is indicated by an orange line. B) PCR verification of the Δ*app1* line. C) Western blot of $APP1^{3xHA}$, Δ*app1*, and $APP1^{Comp}$ strains probed with mouse anti-HA antibodies demonstrating loss of 3xHA protein signal with Δ*app1* and restoration of protein expression with $APP1^{Comp}$. CRC, coding region check. Amplicons of CRC #1, #2, and #3 were 1079 bp, 765 bp, and 1101 bp, respectively. Amplicons of 5' HPT Check and 3' HPT Check were 971 bp and 596 bp, respectively. Molecular weight markers are denoted by kilobase (kb) or base pair (bp) markers.

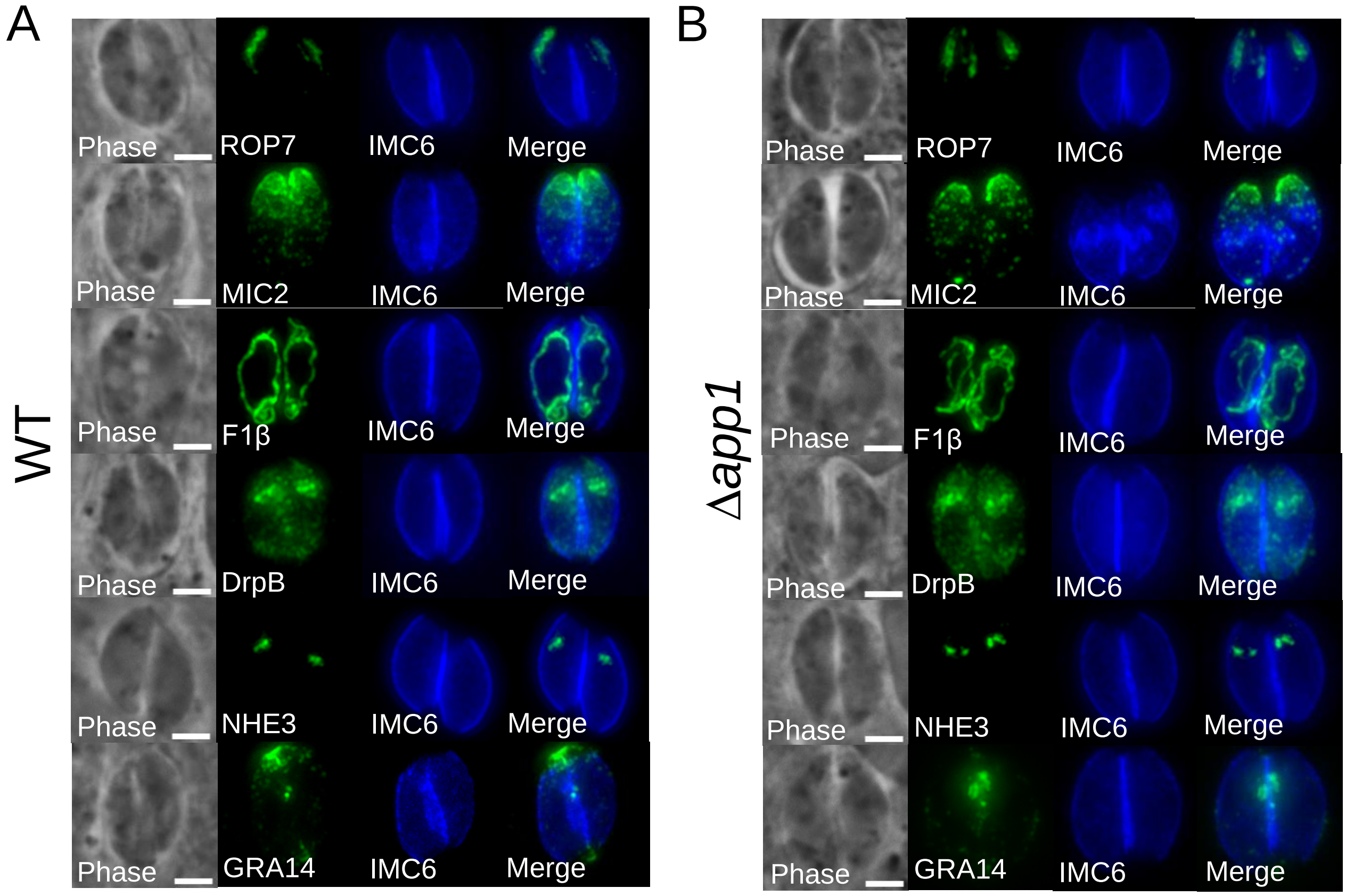

Supplementary Figure 3. Organelle panels of wild-type and Δ*app1* parasite strains. A) Organelle panel of wild-type and B) Δ*app1* parasites probing rhoptries (ROP7), micronemes (MIC2), mitochondrion (F1β ATPase), post-golgi vesicles (DrpB), plant-like vacuolar compartment (NHE3), and dense granules (GRA14). Green, mouse anti-ROP7, mouse anti-MIC2, mouse anti-F1β ATPase, rat anti-DrpB, guinea pig anti-NHE3, mouse anti-GRA14; blue, rabbit anti-IMC6.

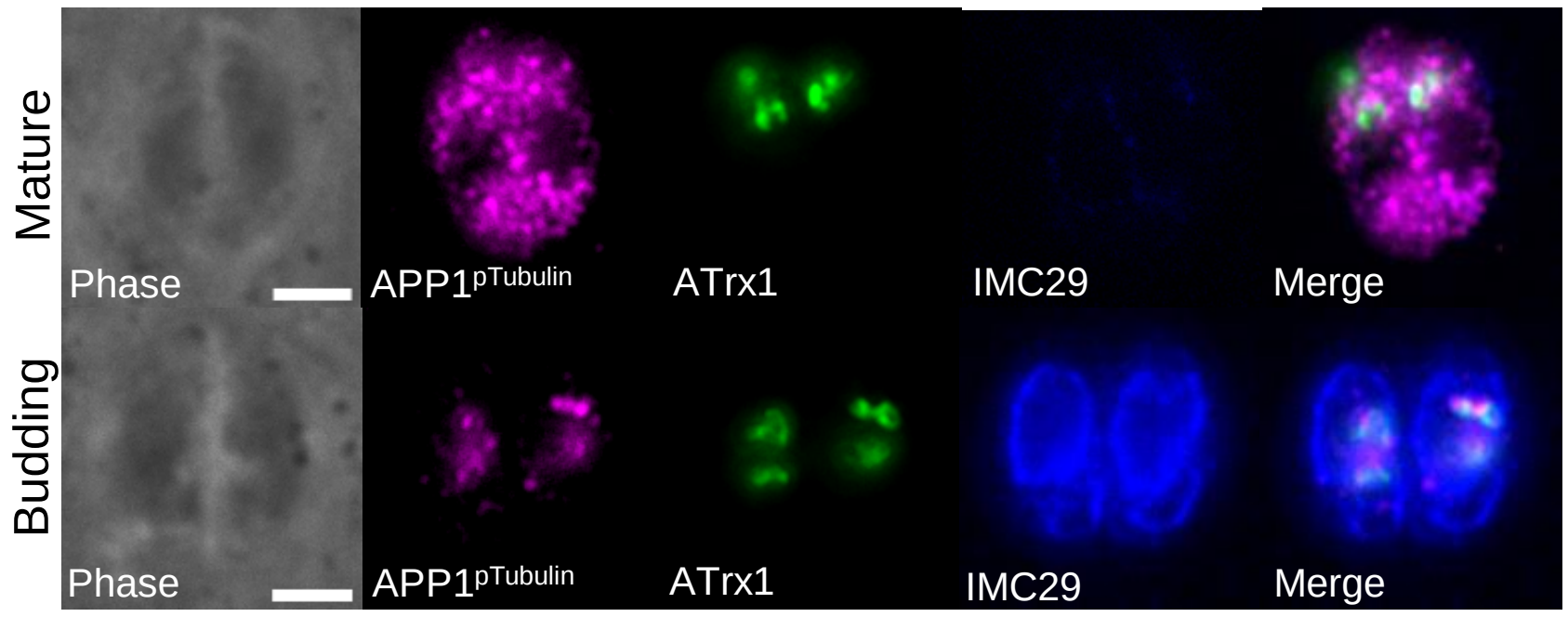

Supplementary Figure 4. The APP1^{pTubulin} strain rescues the apicoplast segregation defect in Δ*app1* parasites. A) IFA images of the APP1^{pTubulin} strain in mature (top) parasites and budding (bottom) parasites. Apicoplast inheritance was fully rescued with complementation of Δ*app1* with APP1^{pTubulin}. In mature parasites, APP1^{pTubulin} signal was diffusely cytoplasmic. In budding parasites, APP1^{pTubulin} localized correctly to the inner face of the daughter IMC.

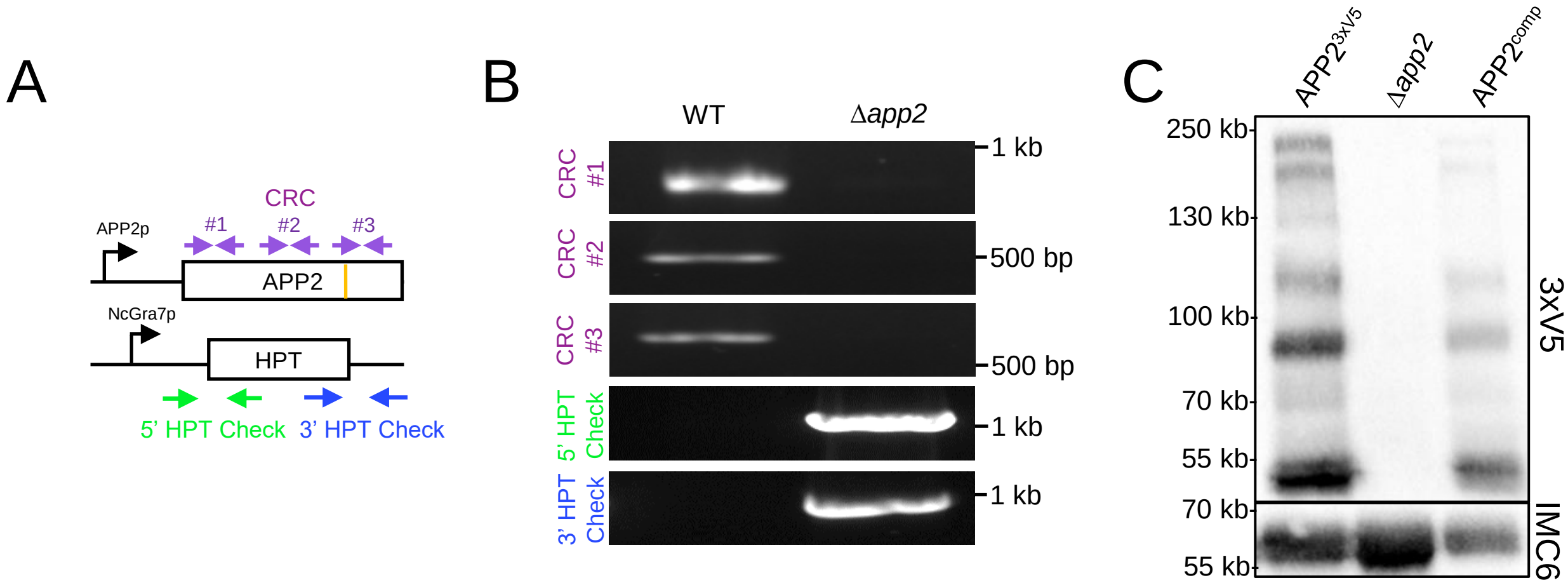

Supplementary Figure 5. PCR verification and western blot of knockout and complemented APP2 strains. A) Schematic of primer design for validation of the Δ*app2* strain. The Cas9 guide sequence from base pairs 2624-2643 is indicated by an orange line. B) PCR verification of the Δ*app2* line. C) Western blot of APP2^{3xV5}, Δ*app2*, and APP2^{comp} lines probed with rabbit anti-V5 antibodies demonstrating loss of 3xV5 protein signal with Δ*app2* and restoration of protein expression with APP2^{comp}. The primary translation product band and multiple breakdown product bands were detected in the APP2^{3xV5} and APP2^{comp} lines. CRC, coding region check. Amplicons of CRC #1, #2, and #3 were 819 bp, 533 bp, and 719 bp, respectively. Amplicons of 5' HPT Check and 3' HPT Check were 1088 bp and 954 bp, respectively. Molecular weight markers are denoted by kilobase (kb) or basepair (bp) markers.

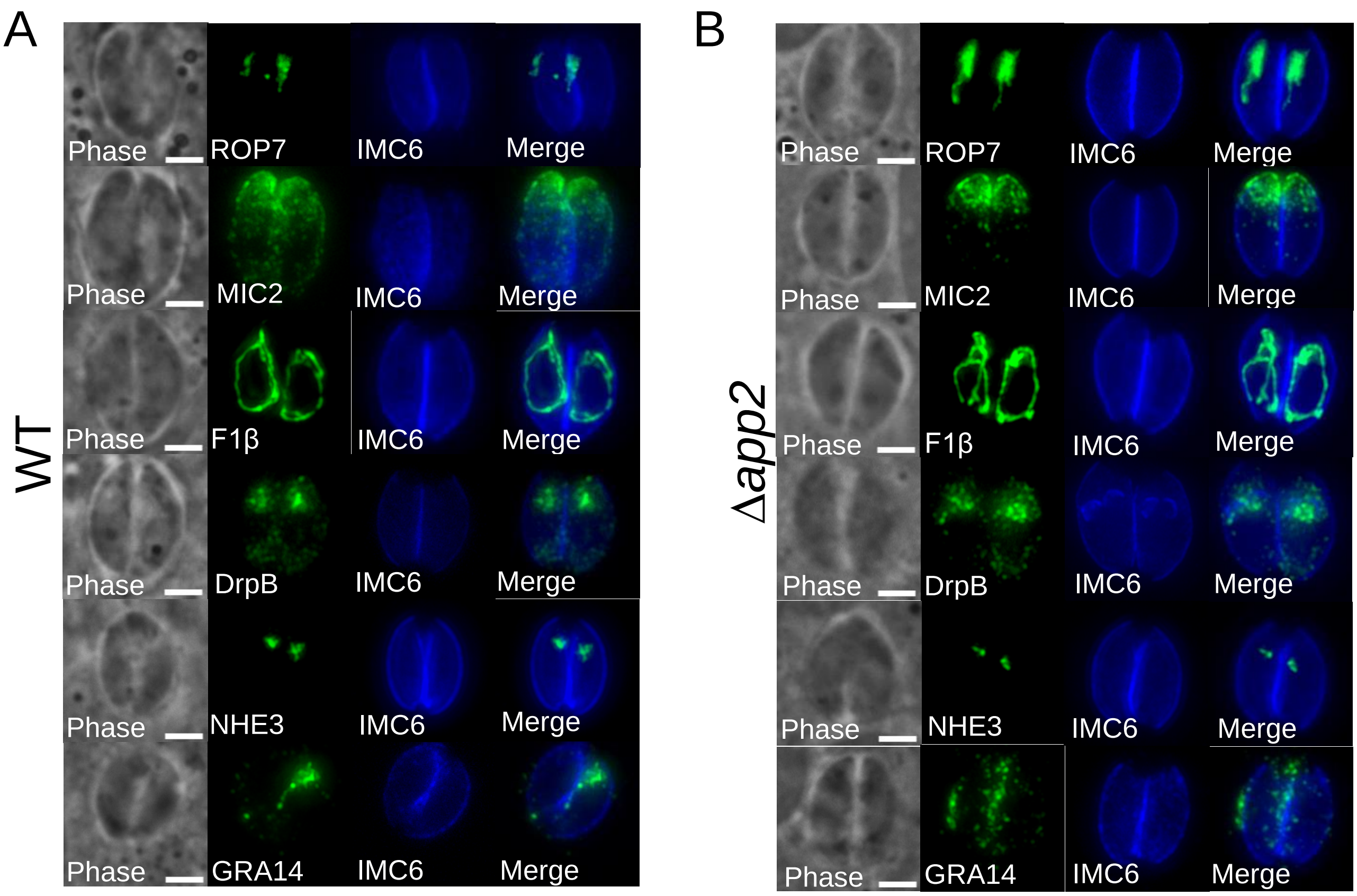

Supplementary Figure 6. Organelle panels of wild-type and Δ*app2* parasite lines. A) Organelle panel of wild-type and B) Δ*app2* parasites probing rhoptries (ROP7), micronemes (MIC2), mitochondrion (F1β ATPase), post-golgi vesicles (DrpB), plant-like vacuolar compartment (NHE3), and dense granules (GRA14). Green, mouse anti-ROP7, mouse anti-MIC2, mouse anti-F1β ATPase, rat anti-DrpB, guinea pig anti-NHE3, mouse anti-GRA14; blue, rabbit anti-IMC6.

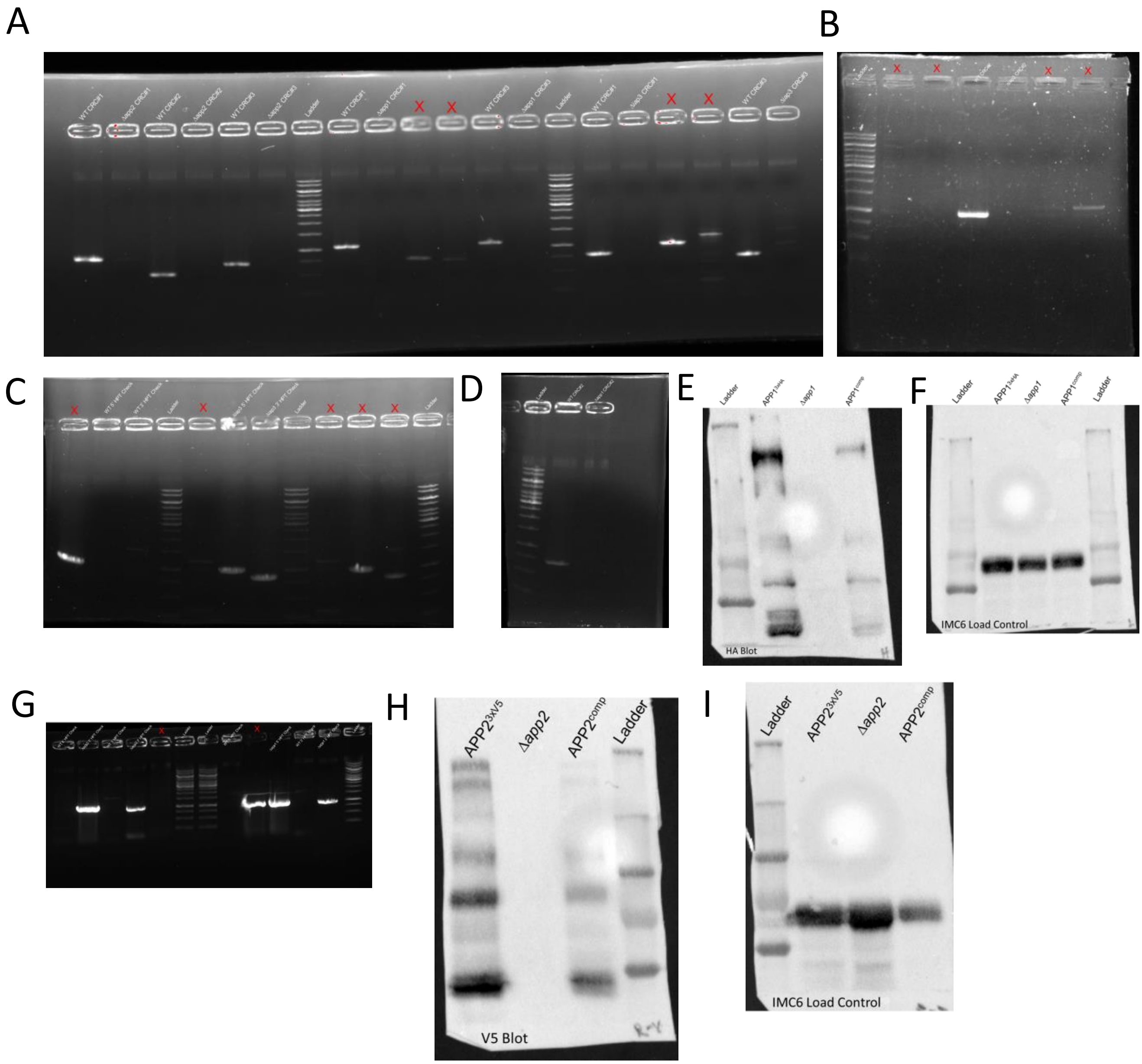

Uncropped blots and gels. A) APP2, APP1, and IAP3 CRC Checks PCR Gel for Supplementary Figures 1b, 2b, and 5b. B) IAP3 CRC#2 Check PCR Gel for Supplementary Figure 1b. C) IAP3 Fwd and Rev HPT Checks for Supplementary Figure 1b. D) APP1 CRC#2 Check PCR Gel for Supplementary Figure 2b. E) APP1 Western Blot HA Blot for Supplementary Figure 2c. F) APP1 Western Blot IMC6 Load Control for Supplementary Figure 2c. G) APP2 and APP1 Recombination Checks PCR Gel for Supplementary Figures 2b and 5b. H) APP2 Western Blot V5 Blot for Supplementary Figure 5c. I) APP2 Western Blot IMC6 Loading Control for Supplementary Figure 5c.
