# Supplementary material for: Identification and characterization of factors that govern apicoplast segregation in Toxoplasma gondii
Source: Nat Commun. 2026 Oct 5;17:10369. doi: 10.1038/s41467-026-78102-x (PMC13639046; doi:10.1038/s41467-026-78102-x)
Supplement: Supplementary file 2 [file 41467_2026_78102_MOESM5_ESM.pdf]

nature portfolio

Peer Review File

Identification and characterization of novel factors that govern apicoplast segregation in Toxoplasma gondii

Corresponding Author: Professor Peter Bradley

This file contains all reviewer reports in order by version, followed by all author rebuttals in order by version.

Version 0:

Reviewer comments:

Reviewer #1

(Remarks to the Author)
The manuscript by Mead and colleagues starts with the characterization of IAP3, a protein that was previously identified in proximity biotinylation experiments where an IMC protein, IMC29, was used as a bait. Although the authors attempt to characterize the IAP3 and find no discernable organellar defects, they fuse the protein to TurboID and perform proximity biotinylation studies. Subsequently, they identify a protein named APP1 (which is the same protein studied by Echlin et al).The authors generate a full knock out strain of APP1, and show that depletion of the protein triggers apicoplast inheritance defects. Interestingly, truncation experiments on the protein reveal that two regions of APP1 are essential for its function: residues 800-140 and the last 350 residues of the protein. Subsequently, the authors employed a proximity biotinylation assay to identify APP1 interactors and identify a novel protein localized in close proximity to APP1, which they name APP2. While APP1 seems to be associated with the daughter cell scaffold, APP2 might be associated with the apicoplast. The authors propose that APP1 and APP2 form a bridge to connect the apicoplast to the centrosome, with APP1 forming a close association with the daughter cell scaffold, and APP2 associating with the apicoplast membrane. Together, this two proteins (along with APP3, characterized in Echlin et al), seem to be three new players involved in the apicoplast segregation machinery in T. gondii and its relatives.
Although the discovery and characterization of APP1 and APP2 undoubtably contributes to understanding the factors involved in apicoplast partitioning, the manuscript has several flaws that hinder its conclusions. As such, it is not ready for publication.
One of the most significant shortcomings in the paper is the fact that the authors decided to generate IAP3, APP1 and APP2 knockout strains. What is the rationale behind this reasoning? All those proteins are predicted to be essential for parasite survival, the authors are aware of it (IAP3 has a phenotype score of -5.06, APP1 score is -4.59, and APP2 is -3.18). Generally, if a protein is deemed to be essential in T. gondii, a conditional knockdown approach should be implemented. Are the authors convinced that their knockout strains are actually complete knockouts? This is especially concerning because they state that the IAP3 parasites eventually revert back to wild-type growth. How can they explain that? More details regarding the genetic modification they implement should be added in Supplementary Figure 1 (where does Cas9 cut? Why is there a new promoter in the locus). The proteins that the authors are studying are large, so how can they be sure that their approach completely eliminated the loci and did not generate truncated—but still functional—versions of IAP3, APP1 and APP2? Perhaps that is why there are parasites able to form plaques in the ΔAPP1 and ΔAPP2 strains.
Another shortcoming in this manuscript is the proximity biotinylation approach. First of all, the images in figure 2B are not really convincing and look almost identical due to the presence of endogenously biotinylated proteins in the apicoplast even in the absence of biotin. Similarly, the streptavidin staining in Figure 7b it appears to be everywhere like in the cell. Perhaps the cells where incubated with biotin for too long? TurboID is known to be a faster version of the original BioID, so what if the rationale for incubating the parasites for 24 hours with biotin? In any case, a western blot will also be more informative for figure 7b.
Finally, most of the results and observations in this manuscript lack quantifications of the phenotypes observed in knockout parasites. It is hard to draw any conclusions when it seems that there is only one representative image. Figures showing disrupted apicoplast morphology during knockout should be supplemented by quantifications of the disrupted apicoplast phenotype (such as the percentage of vacuoles with abnormal apicoplast segregation relative to controls). This is both valid for most IFA observations and the TEM results. The authors might also want to quantify apicoplast morphology during its division to strengthen their results.
Here are also other minor concerns:
- Do the missegregated apicoplasts has abnormal gene expression patterns during knockout of both APP1 and APP2? This

is not discussed in the manuscript.
- The authors should keep the pseudo coloring consistent for each protein across the figures (the colors are inverted in figures 3B and 3C)
- The authors name the protein APP1 just before figure 4C so the labelling in the figures switches from geneID to name, which is confusing. It will be best to see the gene named earlier and a consistent name used throughout the manuscript and figures. The same observation goes for APP2.
• Second panel in figure 2 is cut off
• In the supplementary table, why is does column “287970_K2_vs_287970_Turbo_log2.fold.change” show a negative result? An enrichment should be positive
• There is no raw proximity biotinylation data for the experiments performed with APP2 Turbo ID. Please add them to the corresponding supplementary table.
• The results regarding IAP3 are not dicussed. In fact the first part of the manuscript, where the authors describe and characterize IAP3, seems completely disconnected from the rest of the paper.

Reviewer #2

(Remarks to the Author)
I co-reviewed this manuscript with one of the reviewers who provided the listed reports. This is part of the Nature Communications initiative to facilitate training in peer review and to provide appropriate recognition for Early Career Researchers who co-review manuscripts.

Reviewer #3

(Remarks to the Author)
I co-reviewed this manuscript with one of the reviewers who provided the listed reports. This is part of the Nature Communications initiative to facilitate training in peer review and to provide appropriate recognition for Early Career Researchers who co-review manuscripts.

Reviewer #4

(Remarks to the Author)
This is a thorough and elegant study that characterizes two novel proteins involved in apicoplast division in the obligate intracellular parasite Toxoplasma gondii. The work significantly expands our understanding of the division of this critical and unique organelle, which is a validated drug target. The conclusions presented are well supported by the data. There are a few minor items that could be addressed to tighten what is already a strong manuscript.
1. The paper lacks the protocol for the TurboID assays. In particular, it would be helpful to indicate what control was used.
2. Line 176-177: For how long were the parasites incubated with biotin?
3. While APP1 was expected to be essential, they were able to established a complete knockout. Have the authors monitored transcript levels of APP1 and APP2 in the APP1 knockout? Some discussion about why the gene is not essential as expected and how it contrast to the phenotype seen in the conditional knockdown of the manuscript from Eichlin et al could be useful.
4. The authors noted the presence of amylopectin granules in the APP1 knockouts. This is reminiscent of what is seen in the bradyzoite stage. Have these parasites been monitored for the expression of bradyzoite cyst markers? Some discussion about the relevance or a possible reason for the presence of the granules would be useful.
5. The rationale for their focus on TgGT1_313360 is unclear. Including a table of selected APP1 interactors within the manuscript (based on some specific criteria) might be useful, especially since a few of them are mentioned in the discussion.

Reviewer #5

(Remarks to the Author)
I co-reviewed this manuscript with one of the reviewers who provided the listed reports. This is part of the Nature Communications initiative to facilitate training in peer review and to provide appropriate recognition for Early Career Researchers who co-review manuscripts.

Version 1:

Reviewer comments:

Reviewer #1

(Remarks to the Author)
I appreciate the detailed response for the authors. The additional experiments and quantifications further strengthen their findings, which are also corroborated in the manuscript by Eichlin and colleagues.

Reviewer #2

(Remarks to the Author)
I co-reviewed this manuscript with one of the reviewers who provided the listed reports. This is part of the Nature Communications initiative to facilitate training in peer review and to provide appropriate recognition for Early Career Researchers who co-review manuscripts.

Reviewer #3

(Remarks to the Author)
I co-reviewed this manuscript with one of the reviewers who provided the listed reports. This is part of the Nature Communications initiative to facilitate training in peer review and to provide appropriate recognition for Early Career Researchers who co-review manuscripts.

Reviewer #4

(Remarks to the Author)
The authors have adequately responded to the reviewers' comments and have strengthened the manuscript accordingly.

Reviewer #5

(Remarks to the Author)
I co-reviewed this manuscript with one of the reviewers who provided the listed reports. This is part of the Nature Communications initiative to facilitate training in peer review and to provide appropriate recognition for Early Career Researchers who co-review manuscripts.

Open Access This Peer Review File is licensed under a Creative Commons Attribution 4.0 International License, which permits use, sharing, adaptation, distribution and reproduction in any medium or format, as long as you give appropriate credit to the original author(s) and the source, provide a link to the Creative Commons license, and indicate if changes were made.
In cases where reviewers are anonymous, credit should be given to 'Anonymous Referee' and the source.
The images or other third party material in this Peer Review File are included in the article’s Creative Commons license, unless indicated otherwise in a credit line to the material. If material is not included in the article’s Creative Commons license and your intended use is not permitted by statutory regulation or exceeds the permitted use, you will need to obtain permission directly from the copyright holder.
To view a copy of this license, visit https://creativecommons.org/licenses/by/4.0/

We thank the reviewers for the positive feedback on the paper which highlights this new discovery of apicoplast segregation machinery in *T. gondii*. The reviewers both noted the significance of the work and made insightful suggestions for improvement of the manuscript. We have addressed each of the comments in the point-by-point response below.

Reviewer 1

The manuscript by Mead and colleagues starts with the characterization of IAP3, a protein that was previously identified in proximity biotinylation experiments where an IMC protein, IMC29, was used as a bait. Although the authors attempt to characterize the IAP3 and find no discernable organellar defects, they fuse the protein to TurboID and perform proximity biotinylation studies. Subsequently, they identify a protein named APP1 (which is the same protein studied by Echlin et al). The authors generate a full knock out strain of APP1, and show that depletion of the protein triggers apicoplast inheritance defects. Interestingly, truncation experiments on the protein reveal that two regions of APP1 are essential for its function: residues 800-140 and the last 350 residues of the protein. Subsequently, the authors employed a proximity biotinylation assay to identify APP1 interactors and identify a novel protein localized in close proximity to APP1, which they name APP2. While APP1 seems to be associated with the daughter cell scaffold, APP2 might be associated with the apicoplast. The authors propose that APP1 and APP2 form a bridge to connect the apicoplast to the centrosome, with APP1 forming a close association with the daughter cell scaffold, and APP2 associating with the apicoplast membrane. Together, this two proteins (along with APP3, characterized in Echlin et al), seem to be three new players involved in the apicoplast segregation machinery in T. gondii and its relatives.
Although the discovery and characterization of APP1 and APP2 undoubtably contributes to understanding the factors involved in apicoplast partitioning, the manuscript has several flaws that hinder its conclusions. As such, it is not ready for publication.

We thank the reviewer for their positive comments on the significance of this new mechanistic discovery of apicoplast segregation and address their comments below.

One of the most significant shortcomings in the paper is the fact that the authors decided to generate IAP3, APP1 and APP2 knockout strains. What is the rationale behind this reasoning? All those proteins are predicted to be essential for parasite survival, the authors are aware of it (IAP3 has a phenotype score of -5.06, APP1 score is -4.59, and APP2 is -3.18). Generally, if a protein is deemed to be essential in T. gondii, a conditional knockdown approach should be implemented. Are the authors convinced that their knockout strains are actually complete knockouts?

We typically use both knockdown and knockout approaches as the phenotype scores do not always correlate with essentiality. For example, we have previously successfully disrupted IMC29 (-3.95, PMID: 36622147), IMC6 (-3.19, PMID: 39264987) and IMC1 (-4.0, PMID: 42113865), while ERK7 is essential (-1.53, PMID: 32409604). Thus, each gene must be interrogated individually. In addition, knockdowns are often incomplete which is a caveat when any growth is observed using this approach. To ensure the knockouts are complete, we conducted additional PCR verifications as described below.

This is especially concerning because they state that the ∆IAP3 parasites eventually revert back to wild-type growth. How can they explain that?

Some strains compensate over time such as IMC29 (PMID: 36622147) and GRA17 (PMID:37498952), or presumably very rapidly (IMT1, phenotype score = -4.02, PMID: 40768331). Compensation may be derived from remodeled protein complexes, changes in trafficking, or adaptations from other proteins participating in the pathway. For ∆*iap3* specifically, we repeated the knockout several times and reverified the plaque assay growth defects after we observed the initial occurrence of compensation (these strains also compensated). The precise mechanism of compensation is unknown but this would best be addressed with RNA-seq studies comparing compensated and uncompensated strains, which we plan to do in the future.

More details regarding the genetic modification they implement should be added in Supplementary Figure 1 (where does Cas9 cut? Why is there a new promoter in the locus). The proteins that the authors are studying are large, so how can they be sure that their approach completely eliminated the loci and did not generate truncated—but still functional—versions of IAP3, APP1 and APP2? Perhaps that is why there are parasites able to form plaques in the ΔAPP1 and ΔAPP2 strains.

We have updated the schematics of the primer design for validation of the IAP3, APP1, and APP2 knockouts in supplementary figures 1, 2, and 5 with an orange line indicating the location of the Cas9 guide sequence, and the figure legends were updated to specify where the Cas9 cuts. Importantly, we have also conducted additional PCR verifications of the coding regions of IAP3, APP1, and APP2 to verify that truncated fragments of the genes are not present in their original genomic location or elsewhere. This new PCR verification data has been included in the supplementary figures.

Regarding the Neospora GRA7 promoter driving the selectable marker HXGPRT - we sought to limit Toxoplasma sequences that could be sites of recombination of the selectable marker used for gene knockouts. We believe this improves the efficiency of knockouts in *T. gondii* and have used this strategy in multiple publications previously (e.g. PMID: 42113865, 36622147, 39264987, 40768331). In the resubmission, we have

also commented on this strategy in lines 562-570 in the materials and methods section “Epitope tagging and gene knockout”.

Another shortcoming in this manuscript is the proximity biotinylation approach. First of all, the images in figure 2B are not really convincing and look almost identical due to the presence of endogenously biotinylated proteins in the apicoplast even in the absence of biotin.

For Figure 2B, we have added insets with a dashed white box and corresponding magnified image to highlight the close but distinct staining between APP1 and streptavidin in the (-) biotin control. Similarly, we added insets with a dashed white boxes and magnified image for the (+) biotin images to emphasize the improved colocalization of APP1 protein and streptavidin staining after incubation with biotin. While this is challenging with low abundance proteins close to the apicoplast, the enrichment of IAP3 and the subsequent identification of APP1 in the mass spectrometry dataset demonstrates the functionality of the fusion protein.

Similarly, the streptavidin staining in Figure 7b it appears to be everywhere like in the cell.

We have added new images for Figure 7B which include insets with dashed white boxes and magnified images for this result. For the (+) biotin treatment images, the magnified image now includes an arrowhead indicating the region of daughter IMC biotinylation, and an arrow indicating biotinylation of the maternal basal complex. The images were thresholded to reduce background staining throughout the cell while still highlighting the daughter IMC and maternal basal complex, which are present in addition to the bait protein.

Perhaps the cells where incubated with biotin for too long? TurboID is known to be a faster version of the original BioID, so what if the rationale for incubating the parasites for 24 hours with biotin?

We agree that TurboID is known to be faster and shorter times can be sufficient. However, we have found with other daughter enriched proteins that 24 hours is likely to improve signal to noise ratios in proximity labeling experiments. In addition, it may allow for the accumulation of biotinylated proteins during replication as IAP3 and APP1 are only expressed during stages of daughter bud development. While each approach has caveats, we feel this is effective and has been successful for us in these and other TurboID experiments (PMID: 36622147, 40768331, 39345210)

We have also added more details for the TurboID proximity labeling in a new methods section titled "TurboID Proximity Labeling" in lines 620-625. In addition, a statement on the rationale for a 24-hour biotin incubation period has been included in this new methods section, as well as in lines 176 and 179.

In any case, a western blot will also be more informative for figure 7b.

We have found that streptavidin western blots with low abundance proteins like IAP3 and APP1, which are only present in developing daughter buds, are largely uninformative as the signal is overwhelmed by the endogenous proteins in the apicoplast and the host mitochondria which surrounds the vacuole. Note that we need to do these experiments on intracellular parasites in which only 25% or so have developing daughter buds (whereas extracellular parasites have few to no daughter buds).

However, we are confident that the IAP3 and APP1 TurboID strains are active. Notably, for the IAP3 TurboID strain, the bait itself was the 3rd most enriched hit of the proteomics dataset. For the APP1 TurboID strain, the APP1 bait was also a relatively well-enriched hit. Furthermore, the APP1 IFA shows labeling of the daughter IMC and basal complex as expected from the dynamic localization of APP1 to these locations. Thus, the fluorescence microscopy data and the outcomes of these experiments demonstrate the activity of the biotin ligase.

Finally, most of the results and observations in this manuscript lack quantifications of the phenotypes observed in knockout parasites. It is hard to draw any conclusions when it seems that there is only one representative image. Figures showing disrupted apicoplast morphology during knockout should be supplemented by quantifications of the disrupted apicoplast phenotype (such as the percentage of vacuoles with abnormal apicoplast segregation relative to controls). This is both valid for most IFA observations and the TEM results. The authors might also want to quantify apicoplast morphology during its division to strengthen their results.

We have now included a quantification of apicoplast loss for the APP2 tag, knockout, and complemented strains (see new Fig. 7K), akin to the APP1 quantification previously provided in Fig. 6B. This new data is discussed in lines 352-355. From the TEM images, we have now included quantification of amylopectin granules present in WT, Δ*app1*, and APP1 complemented strains in the new Fig. 5D. This new data is discussed in lines 268-272. Regarding apicoplast morphology, the TEM data demonstrates preservation of

the overall membrane structure and elongation of the organelle, again supporting the defect is in segregation.

Here are also other minor concerns:
- Do the missegregated apicoplasts has abnormal gene expression patterns during knockout of both APP1 and APP2? This is not discussed in the manuscript.

This is an important consideration that we plan to explore in future studies following this initial discovery and characterization of the machinery mediating apicoplast segregation. We feel that interpreting the significance of gene expression patterns of apicoplasts that successfully segregate or are retained in APP1 and APP2 knockout strains will require significant effort that is best left for the next study.

- The authors should keep the pseudo coloring consistent for each protein across the figures (the colors are inverted in figures 3B and 3C)

The pseudo coloring was corrected to be consistent in the figures. For Figure 3C, APP1 was swapped from magenta to green, and IMC29 was swapped from green to magenta. For Fig 7G, APP2 was swapped to magenta from green, and Atrx1 was swapped from green to magenta.

- The authors name the protein APP1 just before figure 4C so the labelling in the figures switches from geneID to name, which is confusing. It will be best to see the gene named earlier and a consistent name used throughout the manuscript and figures. The same observation goes for APP2.

We have designated APP1 and APP2 earlier and modified the text and figures as requested.

- Second panel in figure 2 is cut off

The panel cutoff was corrected.

- In the supplementary table, why is does column "287970_K2_vs_287970_Turbo_log2.fold.change" show a negative result? An enrichment should be positive

The fold-change values were edited to be positive.

- There is no raw proximity biotinylation data for the experiments performed with APP2 Turbo ID. Please add them to the corresponding supplementary table.

We have run proximity labeling studies for IAP3 and APP1, but we have not yet run a proximity labeling study with APP2 fused to TurboID. The raw data for the IAP3 and APP1 TurboID experiments can be found in Supplementary Table 1 and 2, respectively.

- The results regarding IAP3 are not dicussed. In fact the first part of the manuscript, where the authors describe and characterize IAP3, seems completely disconnected from the rest of the paper.

We used the IAP3 protein as a handle to find other targets in the IMC niche and then focus on the APP1 and APP2 apicoplast segregation defects. The precise phenotype of the IAP3 knockout is unclear, potentially due to its compensation. This protein will be the focus of further studies of this interesting niche of the IMC (apicoplast defects were not detected in knockout or knockdown strains).

Reviewer 4

This is a thorough and elegant study that characterizes two novel proteins involved in apicoplast division in the obligate intracellular parasite Toxoplasma gondii. The work significantly expands our understanding of the division of this critical and unique organelle, which is a validated drug target. The conclusions presented are well supported by the data. There are a few minor items that could be addressed to tighten what is already a strong manuscript.

We thank the reviewer for their positive comments on the manuscript and address the minor comments below

1. The paper lacks the protocol for the TurboID assays. In particular, it would be helpful to indicate what control was used.

We have added a section to the materials and methods titled “TurboID Proximity Labeling” in lines 620-625 referring to a comprehensive proximity labelling protocol that we previously published, and included language to emphasize what aspects of our methods were relevant to the biotinylation experiments: “*The proximity labelling experiments were conducted according to the detailed protocol we previously published.*[60] *The TurboID fusion proteins were generated according to the Epitope*

Tagging section of methods. For large scale TurboID labeling, the fusion protein and an untagged strain were incubated in the presence of biotin for 24 hours before streptavidin purification."

We additionally modified the text to clarify the treatment groups for streptavidin pulldown experiments in lines 178-179: *"After verifying the activity of the biotin ligase, we incubated wild-type control parasites and the IAP3$^{TurboID\text{-}3xHA}$ strain with biotin for 24 hours."*

2. Line 176-177: For how long were the parasites incubated with biotin?

We modified the text to include the incubation time (24 hours) with biotin in lines 176-179.

3. While APP1 was expected to be essential, they were able to established a complete knockout. Have the authors monitored transcript levels of APP1 and APP2 in the APP1 knockout? Some discussion about why the gene is not essential as expected and how it contrast to the phenotype seen in the conditional knockdown of the manuscript from Eichlin et al could be useful.

While we have not monitored transcript levels, we have conducted additional PCR verifications of the coding regions of IAP3, APP1, and APP2 to verify the knockouts (see comment above from Reviewer 1 discussing phenotype scores and essentiality). These verifications have been added to the appropriate supplementary figures. Our results with the APP1 knockout are very similar to that seen from Eichlin et al. We have included a statement in lines 449-452 indicating that the conditional knockdown system used by Eichlin et al. and our complete knockout system resulted in the same phenotype. We also conducted our own APP1 knockdown using the auxin-inducible degron system and see the same phenotype - but chose not to include it as the Eichlin et al knockdown and our knockout make a clear case for the same phenotype in knockout and knockdown parasites (and the paper was fairly long as it is).

4. The authors noted the presence of amylopectin granules in the APP1 knockouts. This is reminiscent of what is seen in the bradyzoite stage. Have these parasites been monitored for the expression of bradyzoite cyst markers? Some discussion about the relevance or a possible reason for the presence of the granules would be useful.

We have quantified the frequency of amylopectin granules as requested by reviewer 1 (see new figure 5D). We have also modified the text from lines 268-272 to discuss the quantification of AGs and the rationale for their manifestation: *"Approximately 44% of the knockout parasites contained amylopectin granules, a sign of stress during metabolic changes in tachyzoites, regardless of the presence or absence of an apicoplast (Fig. 5D).[29] This is likely due to mitochondrial dysfunction due to the loss of*

metabolic products synthesized in the apicoplast that are utilized in the mitochondrion (PMID 40492719).[30]" We have not monitored bradyzoite cyst markers, but this will be done in a future study by creating the knockout in type II strain parasites which are more appropriate for cystogenesis studies.

5. The rationale for their focus on TgGT1_313360 is unclear. Including a table of selected APP1 interactors within the manuscript (based on some specific criteria) might be useful, especially since a few of them are mentioned in the discussion.

We have included two tables indicating GWCS and LOPIT information of selected APP1 interactors. Fig 7C displays a selection of previously characterized hits of interest involved in apicoplast segregation, while Fig 7D displays the APP1 bait protein and the new uncharacterized target of interest, TgGT1_313360. We have additionally modified the text (lines 323-330) to discuss the newly included tables and provide more clarity on the rationale for the selection of TgGT1_313360 as a target of interest: "*We then searched the ranked fold-change results by evaluating hits surrounding the $APP1^{TurboID\text{-}3xHA}$ bait protein (log_2 fold change relative to WT: 6.99) and noted the enrichment of proteins that were previously shown to participate in apicoplast inheritance (Fig. 7C).[16] To identify novel uncharacterized proteins that could be involved in apicoplast inheritance, we screened for hits with CRISPR scores more negative than -3 and that lacked LOPIT localization data. Based on these criteria, we identified a hypothetical protein, TgGT1_313360 (log_2 7.93), as an interesting target for further study (Fig. 7D)."*
